# Global Evidence on the Sustainability of Telemedicine in Outpatient and Primary Care During the First 2 Years of the COVID-19 Pandemic: Scoping Review Using the Nonadoption, Abandonment, Scale-Up, Spread, and Sustainability (NASSS) Framework

**DOI:** 10.2196/45367

**Published:** 2025-02-28

**Authors:** Daniela Valdes, Ankit Shanker, Ghofran Hijazi, Daniel Opoku Mensah, Tahir Bockarie, Ioana Lazar, Siti Aishah Ibrahim, Hamid Zolfagharinia, Rob Procter, Rachel Spencer, Jeremy Dale, Armina Paule, Liam Jonathon Medlin, Keerthana Tharuvara Kallottil

**Affiliations:** 1 Department of Computer Science University of Warwick Coventry United Kingdom; 2 Warwick Medical School University of Warwick Coventry United Kingdom; 3 Warwick Manufacturing Group University of Warwick Coventry United Kingdom; 4 Research & Innovation Birmingham Community Healthcare Foundation Trust National Health Service Birmingham United Kingdom; 5 The Alan Turing Institute for Data Science and AI London United Kingdom; 6 Department of Chemistry University of Warwick Coventry United Kingdom

**Keywords:** pandemic, primary care, outpatients, telemedicine, ambulatory care, global health, patient experience, NASSS, clinician-patient relationship, health inequalities, gray literature, PRISMA

## Abstract

**Background:**

The rapid implementation of telemedicine during the early stages of the COVID-19 pandemic raises questions about the sustainability of this intervention at the global level.

**Objective:**

This research examines the patient experience, health inequalities, and clinician-patient relationship in telemedicine during the COVID-19 pandemic’s first 2 years, aiming to identify sustainability factors.

**Methods:**

This study was based on a prepublished protocol using the Joanna Briggs Institute (JBI) methodology for scoping reviews. We included academic and gray literature published between March 2020 and March 2022 according to these criteria: (1) population (any group); (2) concepts (patient experience, clinician-patient relationship, health inequalities); (3) context (telemedicine in primary and outpatient care); (4) excluding studies pertaining to surgery, oncology, and (inpatient) psychiatry. We searched Ovid Medline/PubMed (January 1, 2022), Web of Science (March 19, 2022), Google/Google Scholar (February and March 2022), and others. The risk of bias was not assessed as per guidance. We used an analysis table for the studies and color-coded tabular mapping against a health care technology adoption framework to identify sustainability (using double-blind extraction).

**Results:**

Of the 134 studies that met our criteria, 49.3% (66/134) reported no specific population group. Regarding the concepts, 41.8% (56/134) combined 2 of the concepts studied. The context analysis identified that 56.0% (75/134) of the studies referred to, according to the definition in the United Kingdom, an outpatient (ambulatory care) setting, and 34.3% (46/134) referred to primary care. The patient experience analysis reflected positive satisfaction and sustained access during lockdowns. The clinician-patient relationship impacts were nuanced, affecting interaction and encounter quality. When mapping to the nonadoption, abandonment, scale-up, spread, and sustainability (NASSS) framework, 81.3% (109/134) of the studies referenced the innovation’s sustainability. Although positive overall, there were some concerns about sustainability based on quality, eHealth literacy, and access to health care for vulnerable migrants and the uninsured.

**Conclusions:**

We identified confusion between the concepts of patient experience and patient satisfaction; therefore, future research could focus on established frameworks to qualify the patient experience across the whole pathway and not just the remote encounter. As expected, our research found mainly descriptive analyses, so there is a need for more robust evidence methods identifying impacts of changes in treatment pathways. This study illustrates modern methods to decolonize academic research by using gray literature extracts in other languages. We acknowledge that the use of Google to identify gray literature at the global level and in other languages has implications on reproducibility. We did not consider synchronous text-based communication.

**Trial Registration:**

Open Science Framework 4z5ut; https://osf.io/4z5ut/

## Introduction

Following the World Health Organization (WHO) announcement declaring COVID-19 a pandemic on March 11, 2020, the organization recommended telemedicine as one of the first critical interventions to minimize demands on stretched supplies of personal protective equipment [1,2]. Although telehealth was not new as a delivery mode, there were great expectations particularly around the use of video consultations in this context. In Africa, telemedicine held promise, as it rationalized human resources allowing national or international experts to relay advice to other clinicians [3]; in the United Kingdom, the pandemic was deemed a “burning platform” to propel the UK National Health Service (NHS) toward widespread adoption of video consultations [4]. Most medical specialties responded to the WHO recommendation with rapid changes in service delivery toward telemedicine (both telephone and a new medium—video consultations) in primary and secondary care across the globe.

In the United Kingdom, 15 months into the pandemic, the academic community and political groups raised questions around the sustainability and impacts of the move toward telemedicine, building upon the learning of the past year toward a “new normal,” particularly as social distancing and lockdown measures were removed [5]. A 2021 report by the UK Health and Social Care Committee defined telemedicine as a “welcomed and positive innovation” overall while highlighting concerns by various national organizations on its impact on health inequalities in terms of exclusion (lack of access), quality, and patient safety. The committee reported important consensus from recognized local institutions such as the Health Foundation and the Kings Fund and patient organizations such as National Voices and Healthwatch on the need for additional research to assess the future of telemedicine in a patient-centered way [6].

In previous protocols [7,8], we relayed how prepandemic evidence synthesis identified several barriers and objections that hindered telemedicine uptake, including technology, workload, and confidentiality [9], as well as concerns regarding appropriateness [10]. However, at the outset of the pandemic, these objections were rapidly overcome, supported by major regulatory and financial enablers [11,12]. Given the considerable incentives and support toward the implementation of telemedicine, there are concerns about the risks of losing some of the advantages of this mode of delivery in a postpandemic future [13], particularly once incentives are no longer in place [14,15]. These concerns apply to not only the United Kingdom, Canada, or the United States but also sub-Saharan Africa [16] and Latin America [17] where considerable barriers persist and there has been more limited evidence of uptake.

The purpose of this scoping review was to explore the global evidence (both academic and nonacademic) surrounding the rapid adoption of telemedicine in outpatient and primary care settings during the first 2 years of the COVID-19 pandemic to identify how elements related to patient experience, clinician-patient relationship, and health inequalities support (or take away from) the sustainability of this delivery model.

## Methods

### Protocol

This review was conducted according to an a priori published protocol [8] following the Joanna Briggs Institute (JBI) methodology for scoping reviews, updated guidance, and data extraction guidance [18-20]. This last guidance clarifies that a review can focus on the most relevant section of a document for analysis, without having to review whole studies in scoping reviews [20]. We outlined deviations from the original protocol and the methodology in Multimedia Appendix 1. A key contribution of this study hinges on the methods used to search and extract gray literature across a wider set of countries as to achieve truly global representation.

### Inclusion Criteria

#### Population, Concept, Context Principle

The inclusion criteria used the Population, Concept, Context (PCC) principle. We classified each article against the PCC framework. Namely, for each document, we sought to identify (1) the population group (if any) to which the document referred, (2) the concept to which it referred (patient experience, clinician-patient relationship, health inequalities, or a combination of these), and (3) in which (clinical) context telemedicine was being used (outpatient, primary care, or particular specialties).

#### Population

The review focused on primary care services offered to the general population. Studies focusing on specific population groups or those with particular conditions within a particular country or geographical area were included.

#### Concept

Although the key concept under consideration was the adoption of “telemedicine,” as defined in the Introduction, we narrowed our inclusion criteria to the sustainability of the interventions, focusing on patient experience, health inequalities, and clinician-patient relationship. Telemedicine has been defined as per our protocol [8] using academic literature [21] and the WHO [22], which defines it as “The delivery of health care services, where distance is a critical factor, by all health care professionals using information and communication technologies [...].” Although we focused on telephone and video as communication technologies, we thank an anonymous peer reviewer who identified that text messaging also falls in this category (albeit not included in our review).

#### Context

The context was primary care services provided during the COVID-19 pandemic in any setting or country during the first 2 years of the pandemic (March 2020-March 2022).

#### Types of Sources

This scoping review considered quantitative, qualitative, and mixed methods study designs for inclusion. In addition, systematic reviews, protocols, other documents, and commentaries or opinions were considered for inclusion in the proposed scoping review. These commentaries or other documents might have appeared in peer-reviewed journals or other gray literature such as industry magazines or reports [23,24].

### Search Strategy

This section summarizes our prepublished protocol [8]. We structured this section by first explaining the selection and identification of search terms (both in English and other languages), then how we used those terms to search for academic and gray literature. To note, the PRISMA-ScR (Preferred Reporting Items for Systematic Reviews and Meta-Analyses Extension for Scoping Reviews) [25] was used to structure our review protocol [8].

#### Identifying Search Terms

Our search terms, which were in English, centered around telemedicine, primary care, and COVID-19 and were expanded through a limited search on Ovid Medline and Web of Science. We also sought guidance from University of Warwick librarians, who identified COVID-19 search terms from the National Institute for Health and Care Excellence (NICE) [26]. Additionally, we examined previous telemedicine protocols in general practice [27] and Primary Care Cochrane Library Protocols. For search terms in other languages, we used a 2-step process to identify relevant terms for the included non-English search results. We used Google Translate and engaged in discussions with native speakers to ensure these were accurate translations.

#### Search Approaches

We had 2 distinct approaches depending on whether we were searching for academic or gray literature. With the aforementioned terms, we searched the following academic databases: Ovid Medline (equivalent to PubMed [28]), Web of Science, and Google Scholar. PROSPERO and Cochrane Library were used to inform our design. PROSPERO in particular was used to check whether there was any ongoing review on the topic. All identified search terms and examples of the searches (for academic and gray literature) can be found in Multimedia Appendix 2, as per our previously published protocol [8].

We searched Ovid Medline/PubMed (January 13, 2022), Web of Science (March 19, 2022), Livivo (March 15, 2022), Scopus (March 19, 2022), PROSPERO (January 12, 2022), Cochrane Library (January 12, 2022), and Google/Google Scholar (February 2022 and March 2022).

As shown in Multimedia Appendix 2, to identify relevant gray literature published at the time of the pandemic, we used advanced Google search criteria with simplified search terms. We combined the terms telemedicine, “Primary Care,” and COVID-19 with (1) “patient experience,” (2) “health inequalities,” or (3) “patient-clinician interaction” and asked the search engine to provide pdf-only results within the years 2020 through 2022. Although we recognize it is not fully possible to reproduce Google searches, the selection of pdf documents was aimed at identifying the most retrievable and credible gray literature [8] while, at the same time, supporting reproducibility of the analysis [23,24].

We undertook these searches in English, Chinese, Spanish, Arabic, Portuguese, Hindi, and Indonesian. To improve representation of African countries due to difficulties searching in Urdu, we undertook additional Google searches in English including country-specific results for the 5 largest African countries by population (Nigeria, Ethiopia, Democratic Republic of Congo, Egypt, and South Africa [29]). For Google searches, we selected the first 30 results by relevance, and for English-based results, we selected the top 10 results by country. We selected the first 30 results based on the literature [30-33], timeline, and budget. Further, as can be seen in the example for a failed search for Pakistan in Multimedia Appendix 2, Google searches only provided less than a handful of results when restricting by country of publication.

We followed established guidelines for analyzing non-English text [34]. We used machine translation via Google Translate to translate at least 3 paragraphs containing the key search terms (telemedicine, primary care, and any of the combinations of patient experience, clinician-patient relationship, and health inequalities). We selected 3 paragraphs that were roughly equivalent in the number of words to that of an abstract.

### Screening

The authors used a single-phase, double-blind screening of abstracts and extracts based on the eligibility criteria. Screening instructions were tested by 4 authors across a sample of 50 abstracts to verify the instructions had been properly understood. The remaining articles were allocated across several combinations of pairs of authors using double-blind screening and Rayyan [35] as an aid. Any discrepancies were resolved by the pair of authors themselves and verified by the lead author before data extraction. Given the extended scope of our review, and in agreement with the prepublished protocol and the latest JBI guidance [20], there was no full-text screening as the abstracts and extracts were our main data source. Abstracts and extracts have been made available in Multimedia Appendix 3.

### Data Extraction

The authors used double-blind extraction of data using an Excel table template as outlined in Multimedia Appendix 4. Following the latest JBI guidance for scoping reviews [20], we chose to focus data extraction on abstracts and extracts only, reflecting the wide research design that allowed us to accommodate the breadth of the review in terms of (1) themes; (2) a worldwide, multilanguage approach; and (3) sources. Using a data extraction tool shared in Multimedia Appendix 4, we mapped the text in tabular form against the NASSS framework [36] domains, noting some of the document sections might touch upon 1 or more domains.

### Data Analysis and Presentation

In agreement with the latest JBI methodological guidance [18-20], no critical appraisal was undertaken, and the final presentation of results consisted of the following. In the first section, the results of the search were presented in a PRISMA-ScR flow diagram [25], including a flowchart and a checklist, and we conducted a table analysis of more detailed characteristics of the included documents (Table S1 in Multimedia Appendix 5). We captured the number of articles that included certain data against the PCC framework. Understandably, in the “population” label, the numbers do not add up to the total as some categories are not mutually exclusive. A color-coded (heat map) mapping was created in tabular form against the NASSS framework’s [36] domains: (1) the condition, (2) the technology, (3) the value proposition, (4) adopters, (5) organizations, (6) wider system, and (7) embedding and adaptation over time. The heat map shows graphically the maximum and minimum numbers of references for each domain (using the average counts for double-blind data extraction and mapping). We performed a narrative analysis (including sentiment analysis) of references to the sustainability of video consultations outside of social distancing restrictions brought about by the pandemic. The results are discussed from the point of view of sociotechnical grounded theory, providing strengths and limitations of the sources and the review method itself. We included a statement of positionality in our conclusions, as well as recommendations for research and practice. We believe that reflexivity through researcher positionality is fundamental to decolonizing global health research that seeks to include voices and perspectives usually marginalized from the academic discourse [37-39].

## Results

### PRISMA Study Inclusion

**Figure 1:**
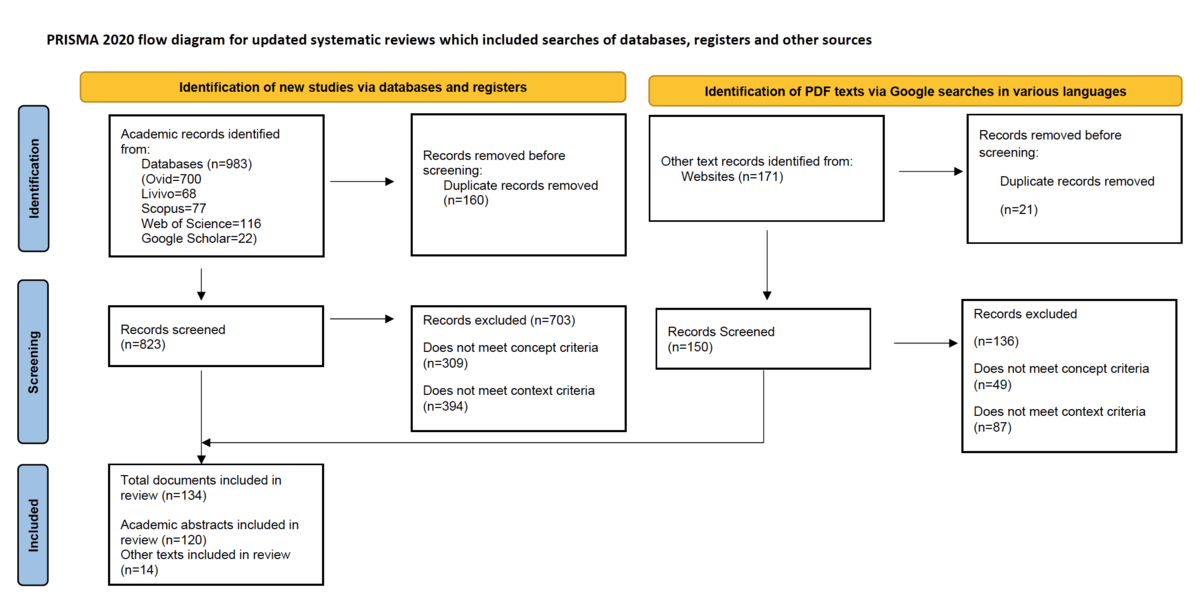
PRISMA-ScR (Preferred Reporting Items for Systematic Reviews and Meta-Analyses Extension for Scoping Reviews) flowchart outlining the process of identification, screening, and final inclusion across various types of data sources.

Academic database searches identified 983 records, and 171 additional records were identified through Google searches for various languages and countries. For Google Arabic and Google Pakistan, there were no results that matched the search criteria. Of the total 1154 articles, 181 were identified as duplicates and were removed: 160 duplicates were academic articles, and 21 duplicates were identified among the nonacademic articles. After the removal of duplicates, there were 973 articles to be screened. Of these 973 articles, 823 articles were from academic databases, and 150 were from nonacademic databases. After the abstract screening and before data extraction, 703 academic and 136 nonacademic articles were excluded, leaving a total of 134 documents [29,40-171] for data extraction including 120 academic articles and 14 extracts from gray literature searches. See the PRISMA-ScR [25] flowchart (Figure 1).

### Summary of Document Types

We separated the academic studies (which we identified as those having a clearly labelled abstract with introduction, aims, methods, results, and discussion sections) from the other documents (or gray literature) and found that these other documents represented 27.6% (37/134) of the selected documents. Searches in academic databases and journals provided a small group of gray literature in the form of commentaries or guidelines. Conversely, general internet searches also identified a minority of academic documents.

The document pool achieved global representation, with documents from all continents, including South America [46,110,166] and Africa [116,121]. There was, however, overrepresentation from North America (58/134, 43%), with a large proportion of documents from the United States.

In terms of the methodology in the selected documents, over 72% (96/134) of the documents used surveys, questionnaires, or interviews. The use of surveys and questionnaires was closely related to the type of design observed, with most studies being cross-sectional (96/134, 72%). Finally, regarding the telemedicine medium, the documents did not specify the medium by generally referring to “telemedicine” in 57% (77/134) of documents. More details are provided in Table S2 in Multimedia Appendix 5.

### Quantitative Classification Against the PCC Framework

**Figure 2:**
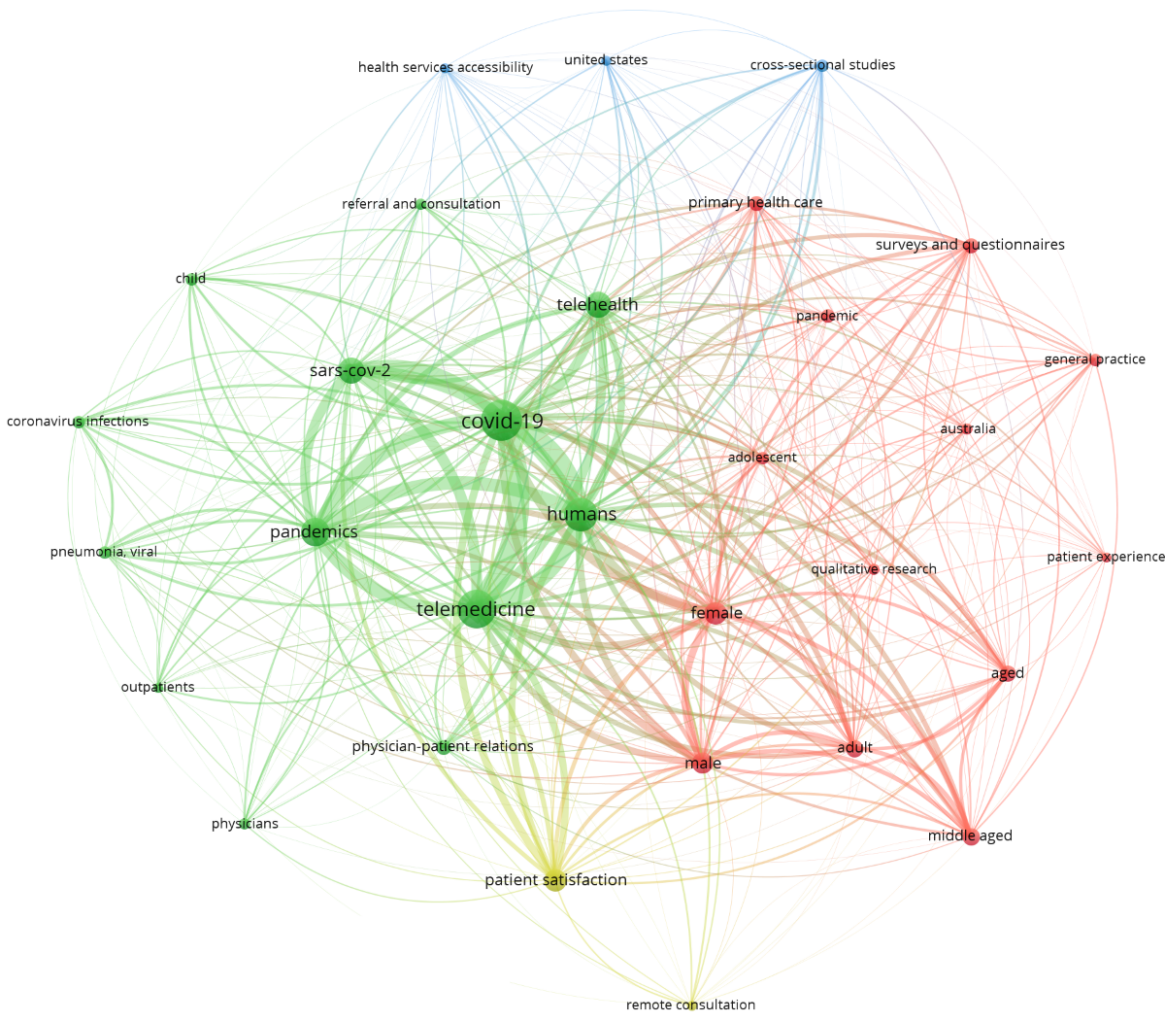
Bibliographic keyword analysis.

Table S3 in Multimedia Appendix 5 includes the characteristics of the 134 documents against the protocol’s PCC. We follow with a short commentary highlighting any documents that exemplify these findings. Table 1 shows how the documents related to the various key concepts explored (clinician-patient relationship, health inequalities, and patient experience), and the following paragraphs summarize the key findings. In addition, Figure 2 shows the results of the bibliographic keyword analysis.

In terms of population groups, a large subset of abstracts and extracts (66/134, 49.3%) reported no specific demographics nor patient characteristics (see, for example, the extract from the board report from the East Kent Hospitals NHS Foundation Trust [64] or Karacabeyli et al [95]). The main demographic characteristics reported in abstracts and extracts were age (39/134, 29.1%) and sex (20/134, 14.9%), with studies also considering sociodemographic factors. It is important to note that a particular document might have tracked more than one characteristic; see, for example, Manski-Nankervis et al [113], which tracked education status, gender, age, and whether patients spoke English at home.

In terms of the concepts studied**,** the most popular concept was the clinician-patient relationship, reported in 28 abstracts. The majority of documents (69/134, 51.5%) combined 2 or 3 concepts. In the following paragraphs, we provide a brief summary of results across the various concepts.

Regarding patient experience, we found 26 studies solely focused on this concept. Patient experience was mostly equated with patient satisfaction and access. There were positive levels of satisfaction overall [99,121,149] and sustained access at the time of lockdowns [48,53].

**Table 1:** Classification of the documents against the key concepts.

Concept		Documents, n
**1 concept**		
	Clinician-patient relationship	28
	Health inequalities	11
	Patient experience	26
**2 concepts**		
	Clinician-patient relationship, health inequalities	11
	Clinician-patient relationship, patient experience	29
	Health inequalities, patient experience	16
**All 3 concepts**		
	Clinician-patient relationship, patient experience, health inequalities	13

Regarding health inequalities, there were only 11 studies solely focused on this concept. We found gaps in evidence that made it difficult to pinpoint the impacts on health inequalities of specific groups, with some evidence of health inequality exacerbation for those considered to be at the fringe of the economic and health system (such as vulnerable migrants, families with high levels of economic deprivation or health care insurance exclusion, or rural communities with limited access to technology) [74,116,140].

Regarding the clinician-patient relationship, there were 28 documents solely focused on this concept, mainly on the clinician-patient interaction and quality of the encounter. There was no consensus regarding whether the impact of telemedicine on the interaction had been positive, neutral, or negative [43,56,70], while there was consensus on the added value of the quality of the remote encounter for triage, follow-up consultations, or chronic condition management [128,133,155].

Finally, concerning the context, we identified 46 documents focused solely on primary care, general outpatient care featured in 19 documents, and various outpatient specialties were featured in 56 extracts.

### Mapping Against the NASSS Framework

**Figure 3:**
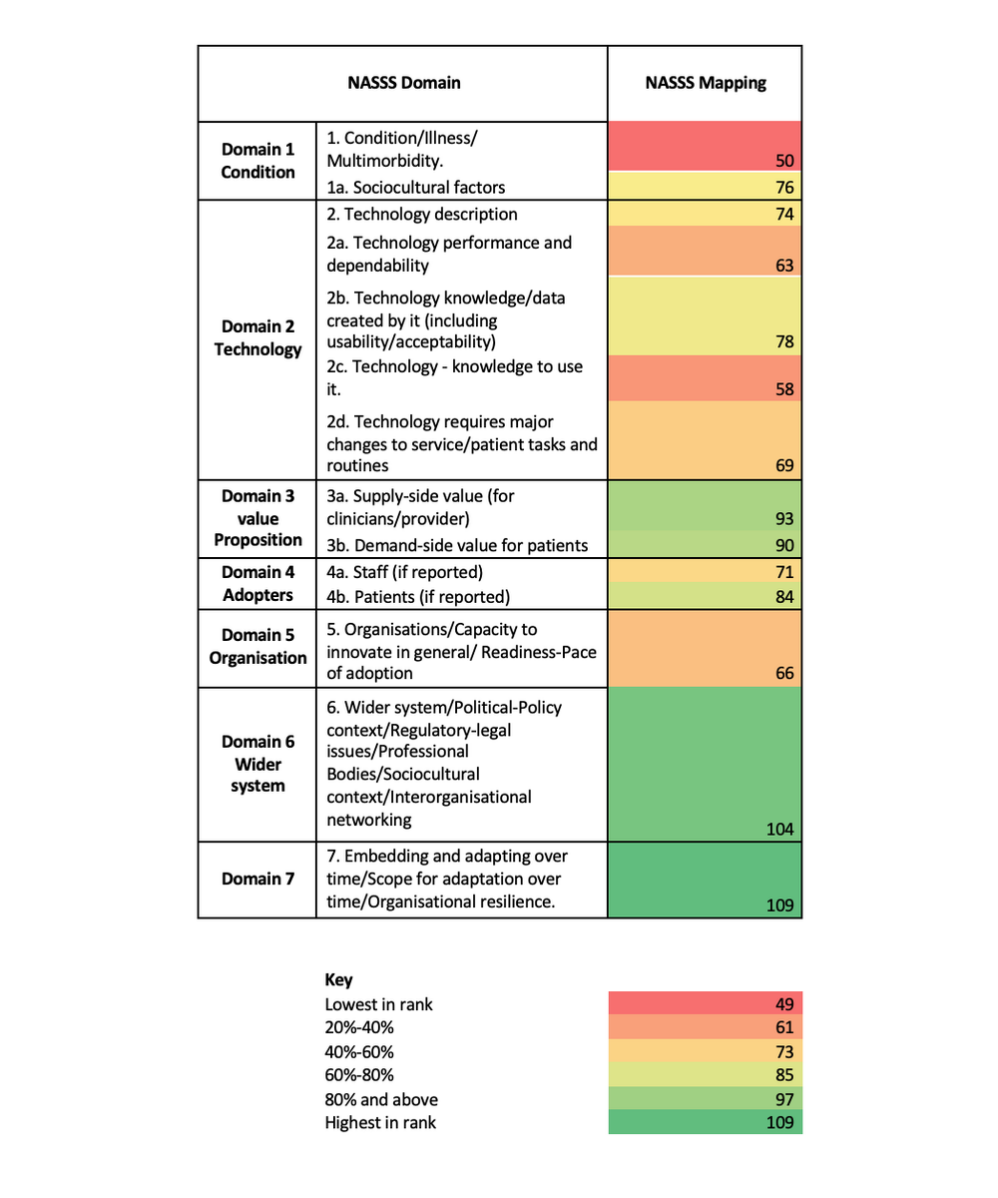
Heat map against each of the nonadoption, abandonment, scale-up, spread, and sustainability (NASSS) domains.

As shown in Figure 3, there was considerable variability in evidence across the NASSS [36] domains and subdomains. Domains 6 (wider system), 7 (time domain), and 3 (value proposition) had the most information. For domain 6, documents referenced the pandemic or pandemic-related lockdowns and infection control measures coupled with regulatory enablers or recommendations (such as mandatory online triage in primary care, parity in payments between face-to-face and telemedicine appointments). There was lower density of information about specific conditions that were being managed with telemedicine (subdomain 1a), knowledge needed to use telemedicine (subdomain 2c), and organizational or implementation aspects (domain 5).

### Narrative Analysis of Sustainability

Roughly over one-half of the documents (75/134, 56%) identified challenges in terms of sustainability or made recommendations on how to address them. Among these, sentiment was mixed or neutral in 40 documents and positive in 31 documents, with only a small subgroup [68,89,141,148] viewing such challenges negatively as barriers to further planning and progress. Challenges and areas of consideration included general planning such as workforce training, digital resources, patient experience, and ethical issues [92] or a more focused look at the technology itself such as more effective digital platforms and increased use of home medical equipment [43]. Other issues such as patient selection were also noted with consideration to disease progression, language and cognitive ability, health literacy, and technology access [40]. The systematic review by Mbunge et al [116] of the digital technologies deployed in South Africa during the pandemic identified a “digital divide” barrier in rural areas and advocated for better networks.

Almost one-third (38/134, 28%) of the documents set out plans for sustainable growth and further embedding, with generally positive sentiment. The report by the East Kent Hospitals NHS Foundation Trust in 2021 [64] mentioned an enhanced engagement plan to meet ambitious targets set by health care authorities for the delivery of telemedicine appointments. Tulupova et al [162] referred to plans for the creation of telemedicine guidelines and an educational program on communications in health care using digital technologies for patients to improve digital health literacy. The remaining documents either did not address the area of sustainability [42,44,49,66,95,101,106,110,111,122,132,143,153,167] or had a generally negative view on the sustainability of the intervention [63,74,80,112,169]. The negative commentary was based on concerns about quality (ie, treating musculoskeletal conditions), eHealth literacy, and access to health care for those at the fringe of the health care system coverage (such as vulnerable migrants and the uninsured).

## Discussion

This section includes 4 main areas: (1) a summary of our findings, (2) a sociotechnical grounded theory research interpretation of the findings (based on Hoda [172]), (3) positioning against the wider and recent literature, and (4) strengths and limitations of our study. The section finalizes with a short conclusion.

### Summary of Findings

Concerning the protocol’s PCC, 49% (66/134) of the documents reported no specific population group targeted by the study (population); according to the UK definition, 55% (74/134) of the studies referred to an outpatient (ambulatory care) setting, and 34% (46/134) referred to primary care (context); and 49% (66/134) of the studies referred to only 1 of the concepts studied (concept).

Mapping to the NASSS framework [36] identified that 93% (125/134) of the studies referenced the sustainability of telemedicine with moderately positive comments.

### Global Representation

We found 134 studies meeting our criteria and achieved global representation. We highlight some of the global results from limited-resource countries here.

Despite the limited evidence for African countries (2 studies [116,121]), our findings were aligned with other African reviews around insufficient evidence of use due to considerable barriers in this region [16] and a lack of “meaningful investment” in this area [173]. We concurred with Nittari et al [174] that several barriers are still present that risk the sustainability of this delivery mode beyond the pandemic.

Studies in South America [46,110,166] provide examples of effective use in outpatient settings (specifically speech and language therapy [46]), as well as reflections on how this new delivery mode in the context of the wider pandemic might have generated an element of mistrust and fear in the clinician-patient relationship [166].

Results from Middle Eastern and Asian countries [41,97,104,105] provide perspectives on the use of telemedicine in orthopedics and hematology while indicating the equivalence of audio and video consultations, an important point to inform telemedicine programs in low-income countries as a way of increasing access to health services.

### Positioning Against the Wider and Recent Literature

This review can be positioned in the emerging literature of reviews around telemedicine during the pandemic period [175-183].

A key finding of our study is how patient experience was generally equated with patient satisfaction. Other studies have found these are often used interchangeably [184-186]. However, accepted definitions of patient experience go beyond satisfaction and “focus on individualized care and tailoring of services to meet patients’ needs” [187]. This is related to another finding, as the studies are mainly observational with no reference to patient experience frameworks, let alone to some emerging frameworks specific to the digital patient experience [173,188]. Our findings on patient experience are aligned with another recently published review focused on the COVID-19 pandemic, with similar categories and findings [178]. In terms of the clinician-patient relationship, our findings were mixed, but recent reviews found that the relationship was “troubled” telemedicine given both patient and clinician reluctance to use [177]. However, others found the tool useful [106].

de Oliveira Andrade et al [175] focused on the role of legislation in the widespread utilization of telemedicine during the pandemic, finding that regulatory frameworks enabled telemedicine spread in areas related to ethics, reimbursement, data safety, and pandemic-related regulatory relaxation (in the United States, for instance, relaxation of interstate practice was particularly relevant [183]). We found a lack of consensus in terms of sustainability as these financial and regulatory incentives dissipated; however, supportive regulation would be a defining factor in its sustainability. Our evidence seems more nuanced than other recent reviews [177,189]. We are aware specialties like family medicine and general practice seem to have a preference for a particular medium, such as telephone instead of video [190], with more work being undertaken regarding the impacts on quality [191] or equivalence between face-to-face and remote consultations [183].

Regarding the impact on health inequalities, another review identified emerging literature on the opinions of vulnerable populations regarding telemedicine [180]. We saw references [192-195] to an emerging framework for digital health equity [196], which we expect will help address the gaps we identified in the design and evaluation of inclusive digital health care services to address the “self-reinforcing effect of digital and social exclusion” [197] and its impact on health care inequalities in access and experience. Our concerns about implications on health inequality resonate with other similar reviews for telemedicine [179].

### Strengths and Limitations

#### Strengths

We mapped the emerging literature (gray and academic) on telemedicine during the pandemic to a well-established framework using 3 lenses (patient experience, health inequalities, and clinician-patient relationship). We identified this as the only systematic review of its kind. Only 1 other review mapped academic literature on video consultations to the NASSS framework [36] but outside the context of the pandemic [37]. Notably, we effectively reflected the experiences of non–English-speaking countries with literature across the 5 continents, so it is truly global health–oriented, with the added values and perspectives of a diverse, multidisciplinary research team.

When identifying the limited literature at the intersection of health inequalities and telemedicine, we provided a brief taxonomy of potential groups impacted differently. Recognizing the multidimensionality and intersectionality of social exclusion, we show that demographic characteristics such as age, sex, and socioeconomic factors have received some attention, but there is still very limited information and not enough to draw solid conclusions on impacts.

From a methodological standpoint, we provided additional insight on how to integrate effectively other documents and nontraditional voices and experiences into academic research with a reproducible approach.

#### Limitations of the Sources

The authors sought gray literature directly via Google to ensure a unified source and methodology to identify and capture experiences from non–English-speaking countries. This is a method with limited reproducibility. Researchers interested in gray literature information in English can consult the UK National Grey Literature Collection Funded by Health Education England. We are aware of additional databases with non-English literature that could be used [198]. Emerging literature has covered mostly single-center survey studies with limited sample sizes, reflective of the immediate experiences arising in the context of the pandemic.

#### Limitations of the Review

We have limited the depth of analysis to accommodate for the extensive scope, in accordance with the JBI guidelines for data extraction [199]. The use of document extracts using the “surrounding keyword” approach needs further development and testing, as we recognize slightly longer extracts are better at conveying enough information to support screening and analysis. We did not consider synchronous text-based communication. The methodology of scoping reviews is still skewed toward evidence from academic publications, which are biased toward researchers from North America and Europe. Although not specified in the current guidance, capping the number of results from traditional academic databases provides a more balanced representation and could reduce duplication while having limited effects on how comprehensive the findings are.

In conclusion, our discussion section has highlighted considerable variation in the emerging literature during the pandemic regarding changes in pathways toward telemedicine. We highlighted the different focus of studies focused on health inequality or outpatient care and the global representation of the studies included (which is a key strength). Of note is the finding equating patient experience with satisfaction, which reflects a potentially limited understanding of sociotechnical views of human-computer interaction and human factors in traditional health service research.

### Conclusions

This section is divided into 3 parts comprising a reflection of our positionality as researchers in analyzing these themes as well as further recommendations for research and practice.

#### Statement of Positionality

Following the method by Pant et al [37], we frame some of our conclusions in the context of our positionality and our aims. We are a diverse group of researchers (with roots in Latin America, the Middle East, Asia, and Africa and with supervisors from the United Kingdom), but our gaze is colonially influenced by our education and current positionality in this country.

By opening our search criteria to non-English documents and gray literature, we succeeded in capturing immediate, emerging experiences at the global level, with 27.6% (37/134) of our documents having no structured abstract and classified as gray literature, with 5 documents from South America and Africa and 13 documents from Asia. The balance, however, is still very skewed toward the United States and English-speaking countries.

Our positionality and knowledge of the UK health system and legislation meant it was difficult to translate these categories to other systems, and we had to modify our parameters and analysis. For instance, although our choice of the UK diversity legislation (“protected characteristics”) as a framework for categorizing health inequalities was helpful, the UK definition of “primary care” was not helpful due to its contrast with the definition set out by the WHO (for example in the work by Dimer et al [46] in Brazil, classifying speech and language therapy services as a “primary health care service,” which are not available in the United Kingdom in this setting).

#### Recommendations for Research

From a methodological standpoint, we urge researchers looking to decolonize academic research to test and evaluate further our approach to using gray literature extracts in other languages, particularly in scoping reviews, as it provides that additional level of immediacy with the phenomenon of study. We noticed confusion on the use of patient satisfaction and patient experience, so research should focus on more robust frameworks reviewing the overall patient pathway (away from the evaluation of a remote encounter [184-186]). As expected, our research found mainly descriptive documents, so future research should focus on robust evaluation of clinical outcomes arising from changes in diagnostic and treatment pathways away from face-to-face settings. We recommend future research with a narrower approach to specific population groups and more focused on access and outcomes (Table S1 in Multimedia Appendix 5). From a sociotechnical research perspective, we recommend future research using modern techniques (natural language processing, data mining, and sentiment analysis) focusing on categories with closer links to human-computer interaction. Following publication, we will publish our data on Open Science Framework [200].

#### Recommendations for Practice

The impact of telemedicine on patient safety is critical to determine the sustainability of this intervention when contrasting it with the under- or overutilization of resources [201]. If not ruling out the continued use of telemedicine, the authors outlined the importance of further research and refinements to the intervention itself. We found that models such as the Dynamic Sustainability Framework [75] might be useful to support learning and adaptation with care toward the potential disenfranchisement of some patient groups.

## Appendix

Multimedia Appendix 1 Deviations from the protocol.

Multimedia Appendix 2 Example searches.

Multimedia Appendix 3 Abstracts-extracts used in the study.

Multimedia Appendix 4 Data collection template.

Multimedia Appendix 5 Summary characteristics of the included studies.

Multimedia Appendix 6 PRISMA-ScR (Preferred Reporting Items for Systematic Reviews and Meta-Analyses extension for Scoping Reviews) Checklist.

## Abbreviations

JBI: Joanna Briggs Institute
NASSS: nonadoption, abandonment, scale-up, spread, and sustainability
NHS: National Health Service
NICE: National Institute for Health and Care Excellence
PCC: Population, Concept, Context
PRISMA-ScR: Preferred Reporting Items for Systematic Reviews and Meta-Analyses Extension for Scoping Reviews
WHO: World Health Organization
