# Supplementary material for: Global Evidence on the Sustainability of Telemedicine in Outpatient and Primary Care During the First 2 Years of the COVID-19 Pandemic: Scoping Review Using the Nonadoption, Abandonment, Scale-Up, Spread, and Sustainability (NASSS) Framework
Source: Interact J Med Res. 2025 Feb 28;14:e45367. doi: 10.2196/45367 (PMC11909490; doi:10.2196/45367)
Supplement: Multimedia Appendix 1 [file ijmr_v14i1e45367_app1.docx]

# Deviations from the protocol

| Area | Deviation | Rationale |
| --- | --- | --- |
|  |  |  |
| Key definitions | Concept: Include outpatient care | Alma Ata Declaration [1] coupled with British-Based definition of Primary Care (General Practice) does not translate directly in a Global Health, hence the need of extending to outpatient/ambulatory care. Multi-disciplinary element now present in general practice implies setting exclusion boundaries at specialty level about surgical procedures, oncology and (inpatient) psychiatry |
| Key definitions | Concept: Patient- Clinician Relationship | Expanded to include the wider clinical relationship |
| Key definitions | Context: Healthcare setting | Include Primary care practice, primary care, family care, ambulatory/outpatient care. Include physiotherapy, dental and pharmacy.  Exclude. As above setting exclusion boundaries at specialty level about surgical procedures, oncology and (inpatient) psychiatry. |
| Screening | Use of a prioritisation template | Given remote team working at different times, DV, AS, IL, DM, TB tested a screening template by reviewing a random selection 50 documents, split between academic abstracts and extracts. AS developed s subsequent conflict resolution protocol to address any discrepancies in inclusion/exclusion decisions across pairs of researchers.  Not all reasons for rejection were included across both types of document, so we calculated the ratio of rejections against each of the criteria and extrapolated to the total. |
| Searches | Urdu searches | We found no results in Urdu, and were unable to find results in English but focused in Pakistan (see Appendix III). |
| Screening | Use of automated prioritization tool | Not used due to logistical considerations. |
| Data extraction | Extraction template reflecting NASSS framework [2] sub-domains | Extraction template developed by IL, DM and DV is included as a multimedia appendix  As a result of IS’ review, we noted that the simplified NASSS framework [2] picture outlined Domain 3 as a ‘supply value proposition’ focused on the developers of the technological tool. When reviewing Greenhalgh and Abimbola (2019) [3] it was noted that Domain 3 also pertains to the wider system. We used this distinction to separate the ‘clinician/provider-based value’ and ‘patient-based’ value, noting there were no references to developers of telemedicine tools among the abstracts/extracts. |
| Data extraction | Double-blind extraction | A second reviewer (DV and AP) scanned 10% of the selected articles at random to identify missing areas; additional review was undertaken as part of the analysis phase, by re-scanning the extracts to confirm the extraction against particular domains. |
| Analysis | Analysis in tabular form | The NASSS framework [2] table is constructed as a ‘heat map’. |

**References**

1. Valdes D, Alqazlan L, Procter R, Dale J. Global evidence on the rapid adoption of telemedicine in primary care during the first 2 years of the COVID-19 pandemic: a scoping review protocol. Syst Rev. Jun 19, 2022;11(1):124.
2. Greenhalgh T, Wherton J, Papoutsi C, Lynch J, Hughes G, A'Court C, et al. Beyond Adoption: A New Framework for Theorizing and Evaluating Nonadoption, Abandonment, and Challenges to the Scale-Up, Spread, and Sustainability of Health and Care Technologies. J Med Internet Res. Nov 01, 2017;19(11):e367.
3. Greenhalgh T, Abimbola S. The NASSS Framework - A Synthesis of Multiple Theories of Technology Implementation. Stud Health Technol Inform. 2019 Jul 30;263:193-204.
