# Supplementary material for: Global Evidence on the Sustainability of Telemedicine in Outpatient and Primary Care During the First 2 Years of the COVID-19 Pandemic: Scoping Review Using the Nonadoption, Abandonment, Scale-Up, Spread, and Sustainability (NASSS) Framework
Source: Interact J Med Res. 2025 Feb 28;14:e45367. doi: 10.2196/45367 (PMC11909490; doi:10.2196/45367)
Supplement: Multimedia Appendix 2 [file ijmr_v14i1e45367_app2.docx]

# Example searches

Please note this section has been taken verbatim from the protocol [1].

#### Search terms and examples

PubMed Search

(((primary care) AND (telemedicine adoption) AND (pandemic)) AND ((patient experience) OR (health inequalities) or (patient clinician relationship))) AND ((systematic review[Title]) OR (scoping review[Title]))

PROSPERO SEARCH

(telemedicine AND (primary care)) AND (((coronavirus or corona-virus) AND (wuhan or beijing or shanghai or Italy or South-Korea or korea or China or Chinese or 2019-nCoV or nCoV or COVID-19 or Covid19 or SARS-CoV* or SARSCov2 or ncov)) OR (pneumonia AND Wuhan) or "COVID-19" or "2019-nCoV" or "SARS-CoV" or SARSCOV2 or 2019-nCov or "2019 coronavirus" or "2019 corona virus" or covid19 or ncov OR "novel corona virus" or "new corona virus" or "nouveau corona virus" or "2019 corona virus" OR "novel coronavirus" or "new coronavirus" or "nouveau coronavirus" or "2019 coronavirus") AND (general_interest OR Health inequalities/health equity OR International development OR Public health including social determinants of health):HA NOT Animal:DB.

#### Simplified search terms for other languages

**Top five internet languages**

- **English:** telemedicine, “primary care”, “patient experience”, “health inequalities”, “patient-clinician trust”
- **Chinese** (simplified): 远程医疗, 初级保健, 患者体验,
- **Spanish:** Telemedicina, “atencion primaria”, “experiencia del paciente”, “inequidad en salud”, “confianza en el profesional de la salud”
- **Arabic**: " “عدم المساواة الصحية “التطبيب عن بعد”, “الرعاية الصحية الأولية”, تجربة المريض, “ثقة الطبيب المريض”
- **Portuguese (Brazil)** Telemedicina, “atenção primária a saúde”, “experiência do paciente”, “desigualdade na saúde”, “confiança entre paciente e medico”

**First language spoken in the most populated countries in the world (if not in the above list)**

- Hindi (India): टेलीमेडिसिन, पारिवारिक चिकित्सा, “रोगी अनुभव”, “स्वास्थ्य असमानता”, “रोगी और चिकित्सक के बीच विश्वास”
- Indonesian: telemedicine, “kedokteran keluarga”, “pengalaman pasien”, “ketimpangan Kesehatan”, “kepercayaan antara pasien dan dokter”
- Urdu (Pakistan):” مریض اور معالج کے درمیان اعتماد ”ٹیلی میڈیسن” “ ,“بنیادی دیکھ بھال”, مریض کا تجربہ”, “صحت کی عدم مساوات”,

**Google Search Pakistan (.pk)**

6 March 2021

Terms: **covid-19 "بنیادی دیکھ بھال" "ٹیلی میڈیسن" did not provide any search results when restricting to Pakistan (as not possible to have language search in Urdu)**

When changing to: covid-19 ٹیلی میڈیسن, بنیادی دیکھ بھال filetype:pdf and expanding to ‘any region’ the documentation was mostly US-based.

**Third approach:**

covid-19 "Telemedicine" "Primary Care" site:.pk filetype:pdf

Documents in English but published in Pakistan.

| Number | Link | Title | Extracts |
| --- | --- | --- | --- |
| 1 | https://storage.covid.gov.pk/new_guidelines/01June2020_20200530_Guidelines_for_working_of_OPDs_for_Routine_Patients.pdf | Guidelines for working of Outdoor Patient Departments/ Primary Health Care Centre in wake of COVID-19 outbreak | The activation of services for non-COVID19 patients at primary care would potentially result in increased patient turn over at these facilities but nearer to the doorsteps of population, resulting in lesser mobility of the public and reduction of influx to major hospitals. This guidance reflects the need to 1. Ensure provision of services for illnesses other than COVID19 in OPDs of large hospitals and linking the primary care doctors with the specialists at hospitals through telemedicine where feasible (preferably all facilities to be linked through internet) 2. Identify persons with presumptive COVID-19 disease and implement a triage procedure to assign appropriate levels of care through implementation of ‘Fever clinics’ 3. Reduce the burden on emergency departments and major hospital OPDs so that they can deal with COVID-19 cases and serious emergencies 4. Put in place precautionary measures for the doctors/HCPs, all of whom should go through relevant trainings programs (“We Care” and others) Outpatient department All large hospitals offering OPD service to non COVID19 patients and PHCC should develop local SOPs, in consultation with stakeholders, appropriate to their local environment but keeping in mind the general principals stated in these guidelines. Essential functional components of any OPD service should include Screening area, Registration area, Waiting area, Consultation rooms and other services like Pharmacy/labs etc |
| 2 | http://www.na.gov.pk/uploads/1591967303_200.pdf | Pakistan Annual Plan 2020-21 | Strengthen the official COVID-19 website as the official and trusted source of all information with latest COVID-19 related national statistics, information on testing centres and quarantine facilities, latest guidelines, advisories and other important decisions as well as information from the provinces and live reporting by cities into the dashboards for correct updates; with appropriate social media marketing and dissemination by mainstream media channels, including addressing latest misinformation and myths around the pandemic • Exploit the mediums of radio, television, telemedicine, free SMS help lines and WhatsApp groups to communicate vetted public health messages around COVID-19 and redirect public to official website for verified information • Develop national emergency risk communication and community engagement strategies • Use electronic, social and print media to communicate technically vetted public health messages • Train HRH in emergency health care/ambulatory care, health management and other specialties as identified in the provincial and national health strategic frameworks • Use digital technology & enhance broadband coverage for telemedicine |
| 3 | https://pshealthpunjab.gov.pk/Upload/Downloads/hm3hq3nx.pl020200515.pdf | GENERAL GUIDELINES FOR HOME ISOLATION OF COVID-19 PATIENTS | STEPS TO FOLLOW i. Patients and household members should be educated about personal hygiene, basic IPC measures and how to care as safely as possible to prevent the infection from spreading to household members and contacts. ii. All persons to be Home-Isolated should be provided written instructions regarding monitoring of symptoms and establishing contact with healthcare provider via telemedicine. A. PLACEMENT i. Place the person in a well-ventilated single room (ie, open windows and an open door) preferably with attached bathroom. ii. Ensure that shared spaces (ie, kitchen, bathroom, lounge) are well ventilated (keep windows open) |
| 4 | https://www.aimc.edu.pk/Publication/Vol%2018%20Issue%2004.pdf | COVID-19 OUTBREAK: ITS PSYCHOLOGICALAND BEHAVIORAL EFFECTS IN PAKISTAN | Abstract Background: COVID-19 was declared a pandemic in March 2020 by WHO. With the constant rise in the number of cases worldwide and the increasing number of deaths, COVID-19 is a threat to the psychological wellbeing of the people. Data is needed to form evidence-based strategies to cater to the psychological needs of the people Objectives: This study aims to assess the impact of the pandemic on the mental health of the people and to identify their major concerns regarding the current situations. So that our findings can be used to form future strategies to provide adequate psychological aid to the people of Pakistan. Methodology: From 10th April to 14th April we conducted an online survey to assess the initial psychological response of people of Pakistan to the pandemic. We used the snowball sampling strategy to collect the data. We collected information on demographic data, major concerns of the people, state of awareness about telemedicine facilities, and psychological responses to the spread of misinformation and practices like panic buying. The psychological well-being of people was assessed using the DASS-21 scale for anxiety, stress and depression. Results: Out of our 906 respondents, 40% of respondents reported moderate to extremely severe symptoms of depression, 36.2%% of respondents showed symptoms of moderate to extremely severe anxiety, and 19.9%% of respondents were moderate to extremely severely stressed. Women, young adults (age 18-24), people who had a history of contact with a confirmed or suspected case of COVID-19, and people who showed symptoms like cough, fever, and shortness of breath had comparatively higher DASS scores. The spread of misinformation and practices like panic buying also showed to harm the psychological health of people. 47.4% of respondents were unaware of any telemedicine facility. The majority of the respondents identified WhatsApp as the major source of misinformation 71.1% of respondents said that they would want professional advice on how to cope with the stress of the pandemic. Conclusion: During the initial stages of the pandemic in Pakistan, more than one-third of the respondents reported their psychological effect as moderate to extremely severe. Our findings suggest that women, people with contact history, adults from ages 18 to 24, and people who show symptoms like cough, fever, etc. are high-risk groups. Moreover, the spread of misinformation, and practices like panic buying are associated with poor psychological health. Our findings identify the major concerns of the general population of Pakistan and can be used to formulate future policies. Keywords: Pandemic, psychological impact, COVID-19, mental health, concerns, anxiety, depression, stress. |

#### OVID (Medline all) - Search conducted on 13 January 2022

| Search | Query (title, abstract, keyword heading) | Records retrieved |
| --- | --- | --- |
| #1 | telemedicine or telehealth or "digital health" or phone or telephone or video or virtual or remote or e-consults or e-consultation or tele-consult or phone consult or ehealth or tele-health or tele-medicine or e-health | 371,957 |
| #2 [206] | coronavirus* or coronovirus* or coronavirinae* or Coronavirus* or Coronovirus* or Wuhan* or Hubei* or Huanan or "2019-nCoV" or 2019nCoV or nCoV2019 or "nCoV-2019" or "COVID-19" or COVID19 or "CORVID-19" or CORVID19 or "WN-CoV" or WNCoV or "HCoV-19" or HCoV19 or CoV or "2019 novel*" or Ncov or "n-cov" or "SARS-CoV-2" or "SARSCoV-2" or "SARSCoV2" or "SARS-CoV2" or SARSCov19 or "SARS-Cov19" or "SARSCov-19" or "SARS-Cov-19" or Ncovor or Ncorona* or Ncorono* or NcovWuhan* or NcovHubei* or NcovChina* or NcovChinese* or pandemic or outbreak or epidemic | 398,362 |
| #3 | "general practice" or "general practitioner" or "general physician" or "general clinician" or "general doctor" or "general nurse" or "general nursing" or "general medicine" or "family practice" or "family practitioner" or "family physician" or "family clinician" or "family doctor" or "family nurse" or "family nursing" or "family medicine" or "Primary Care clinic" or "primary care practitioner" or "primary care physician" or "primary care clinician" or "primary care doctor" or "primary care nurse" or "primary care nursing" or "primary care medicine" or "Primary health care clinic" or "primary health care practitioner" or "primary health care physician" or "primary health care clinician" or "primary health care doctor" or "primary health care nurse" or "primary health care nursing" or "primary health care medicine" or "primary healthcare clinic" or "primary healthcare practitioner" or "primary healthcare physician" or "primary healthcare clinician" or "primary healthcare doctor" or "primary healthcare nurse" or "primary healthcare nursing" or "primary healthcare medicine" or GP, doctor, nurse, physician, clinician | 100,210 |
| #4 | #1 AND #2 AND 3 | 318 |
| #5 [207] | "patient experience" or "patient satisfaction" or "patient opinion" or "PREM", or "patient survey" or "patient preference" or "client experience" or "client preference" or "service user preference" or "client experience survey" or "healthcare survey" or "healthcare evaluation" or "health surveys" or questionnaire or survey or "patient voice" or "service user voice" or "patient perspective" or "patient centered quality" or "patient perception" or "patient view" | 1,019,204 |
| #6 [207] | "Health inequalities" or "health inequality" or "health inequity" or "health inequities" or "socioeconomic determinants" or "social determinants of health" or "social disparities" or "vulnerable population" or "disadvantaged populations" or "low income populations" or "ethnic minority" or "indigenous populations" or "underserved populations" or "health inequities" or "geographical inequalities" or "remote populations" or "deprived community" or "social deprivation" or "protected characteristics" or gender, age, "maternity status" or LGBTQ+ or veteran or transgender or "minoritised populations" or disabled or "learning disability" or "married" or "religious populations" or refugee or migrant | 134,379 |
| #7 | "patient trust" or "trust in clinician" or "trust in physician" or "trust in nurse" or "trust in medical professionals" "trust in health practitioner" or "trust in medical profession" or "trust in medical decision making" or "physician-patient Relationship" or "patient-clinician relationship" or "patient clinician communication" or "patient clinician trust" or "patient clinician rapport" or "patient clinician relationship" or "physician patient relationship" or "patient rapport" or "rapport with patient" or "doctor patient relationship", "clinician patient relationship" or "nurse patient relationship" or "doctor patient rapport" or "patient clinician interaction" or "patient clinician encounter" or trust or reliance or confidence | 640,538 |
| #8 | #5 OR #6 OR #7 | 1,677,187 |
| #9 | #4 AND #8 | 128 |
| Limited to 2020 onwards | | 116 |

**References**

1. Valdes D, Alqazlan L, Procter R, Dale J. Global evidence on the rapid adoption of telemedicine in primary care during the first 2 years of the COVID-19 pandemic: a scoping review protocol. Syst Rev. Jun 19, 2022;11(1):124.
2. (NICE. NICE; 2020 Mar. Interim process and methods for developing rapid guidelines on COVID-19 Internet [accessed 2020-10-28] [https://www.nice.org.uk/process/pmg35](https://eur01.safelinks.protection.outlook.com/?url=https%3A%2F%2Fwww.nice.org.uk%2Fprocess%2Fpmg35&data=05%7C02%7CAnkit.Shanker%40warwick.ac.uk%7Cdc19bf3c4280470f69ce08dd41fa5713%7C09bacfbd47ef446592653546f2eaf6bc%7C0%7C0%7C638739266478968927%7CUnknown%7CTWFpbGZsb3d8eyJFbXB0eU1hcGkiOnRydWUsIlYiOiIwLjAuMDAwMCIsIlAiOiJXaW4zMiIsIkFOIjoiTWFpbCIsIldUIjoyfQ%3D%3D%7C0%7C%7C%7C&sdata=WLmtXcradpXDv4SLY9HRi9uN2y9y6sqgZLwtsWbrUD4%3D&reserved=0))
