# Supplementary material for: Global Evidence on the Sustainability of Telemedicine in Outpatient and Primary Care During the First 2 Years of the COVID-19 Pandemic: Scoping Review Using the Nonadoption, Abandonment, Scale-Up, Spread, and Sustainability (NASSS) Framework
Source: Interact J Med Res. 2025 Feb 28;14:e45367. doi: 10.2196/45367 (PMC11909490; doi:10.2196/45367)
Supplement: Multimedia Appendix 5 [file ijmr_v14i1e45367_app5.docx]

# Summary Characteristics of Included Studies

Table S1. Detailed characteristics of the included studies.

| **Author year** | **Population** | **Concept** | **Context (Primary/Outpatient care specialty)** |
| --- | --- | --- | --- |
| East Kent Hospitals NHS Foundation Trust 2021 [1] | Not specified - assume the general population | Clinician-Patient relationship  Patient Experience | Primary care and outpatient |
| European Union 2020 [2] | Not specified - assume the general population | Health inequalities | Primary care and outpatient (also hospital care and social care) |
| Karacabeyli et al. 2020 [3] | Not specified - assume the general population | Health Inequalities Patient experience | Substance misuse |
| Royal College of General Practitioners 2020 [4] | Elderly patients in nursing homes | Health inequalities (also doctors returning to the workforce) | Primary Care |
| National Committee for Quality Assurance (NCQA) 2020 [5] | (Minority) ethnic communities | Clinician-Patient relationship  Health Inequalities | Primary Care |
| Ruhm (Editor) 2021 [6] | Socioeconomic population groups | Health inequalities | Primary Care |
| Mbunge et al. 2022 [7] | Urban/Rural | Health Inequalities | Various (including teledermatology, teleradiology, telecardiology, teleophthalmology, teleneurology, telerehabilitation, teleoncology) |
| Jonnagaddala et al. 2021 [8] | Not specified - assume general population | Clinician-Patient relationship  Patient experience  Health inequalities | Primary Care |
| Philips UK 2021 [9] | Socially deprived population groups | Health Inequalities | Primary Care |
| Li et al. 2022 [10] | Not specified - assume general population | Clinician-Patient relationship | Primary Care |
| Tulupova et al. 2021 [11] | Digital health literate/illiterate population groups | Clinician-Patient relationship  Patient Experience | Primary Care |
| Dimer et al. 2020 [12] | Children and adults | Clinician-Patient relationship  Health Inequalities | Speech and language therapy |
| Hardcastle and Ogbogu 2020 [13] | Urban/Rural | Clinician-Patient relationship  Health Inequalities | Primary Care |
| Bergman et al. 2020 [14] | Not specified - assume general population | Clinician-Patient relationship | Primary care |
| Triantafillou 2020 [15] | General population split by demographic characteristics | Patient experience | Otolaryngology |
| Srinivasan 2020 [16] | Not specified - assume general population | Clinician-Patient relationship | Primary Care |
| Belenkiy 2020 [17] | Not specified - assume general population | Patient experience | Specialized trauma care |
| O'Neill et al. 2022 [18] | Not specified - assume general population | Clinician-Patient relationship | Outpatient care |
| Hofmann et al. 2022 [19] | Maternity status (pre/post natal) | Clinician-Patient relationship  Health Inequalities | Maternity care |
| Manski-Nankervis et al. 2022 [20] | General population split by education status, gender, age, and whether they speak English at home. | Patient experience | Primary Care |
| Mostafa and Hegazy 2020 [21] | Not specified - assume general population | Patient experience | Dermatology |
| Ball et al. 2022 [22] | Not specified - assume general population | Clinician-Patient relationship  Patient experience  Health inequalities | Gynecology |
| Sharma et al. 2022 [23] | High/low video adopters | Clinician-Patient relationship | Ambulatory care |
| Grens et al. 2022 [24] | Not specified - assume general population | Clinician-Patient relationship  Patient Experience | Sexual Medicine |
| Aliberti et al. 2022 [25] | Elderly patients | Clinician-Patient relationship  Health Inequalities | Primary Care |
| Rego et al. 2021 [26] | Not specified - assume general population | Clinician-Patient relationship  Patient Experience | Outpatient care |
| Swaminathan et al. 2022 [27] | Not specified - assume general population | Patient experience | Otorhinolaryngology |
| Heyck et al. 2022 [28] | Patients with particular condition, split by age, ethnicity and fluency in English | Clinician-Patient relationship  Patient experience  Health inequalities | Nephrology |
| Olszewski et al. 2021 [29] | Not specified - assume general population | Patient experience | Neurology |
| Kling et al. 2021 [30] | Not specified - assume general population | Clinician-Patient relationship  Patient Experience | Neurology |
| Taylor et al. 2022 [31] | Urban/Suburban | Clinician-Patient relationship  Patient experience  Health inequalities | Primary care |
| Tornero-Molina et al. 2020 [32] | Patients with a particular condition, split by gender, age, and education level | Clinician-Patient relationship  Patient Experience | Rheumatology |
| Hays and Skootsky 2022 [33] | General population split by gender, age, ethnicity | Patient experience | Ambulatory care, including primary care and specialist physician visits |
| Thiele et al. 2022 [34] | Not specified - assume general population | Patient experience | Rheumatology |
| Volandes et al. 2022 [35] | Older adults, racial and ethnic minority individuals split by age and gender | Clinician-patient relationship | Ambulatory care |
| Nguyen et al. 2022 [36] | General population split by socioeconomic level, race/ethnicity, language and smartphone use | Health Inequalities Patient experience | Safety-net health systems |
| Al-Iede et al. 2021 [37] | Children with a particular condition | Health Inequalities Patient experience | Emergency Department |
| Dempsey et al. 2022 [38] | Children and their families | Health Inequalities Patient experience | Pediatric Gastroenterology |
| Parsons et al. 2022 [39] | Not specified - assume general population | Clinician-Patient relationship  Patient Experience | Neuropsychology |
| Fu et al. 2022 [40] | Vulnerable migrants/asylum seekers, younger adults across various socio-economic conditions. | Health Inequalities | Primary care |
| Sugarman et al. 2021 [41] | General patients and those with (more severe) psychatric conditions | Clinician-Patient relationship  Health Inequalities | Substance misuse |
| Johnson et al. 2021 [42] | Not specified - assume general population | Clinician-Patient relationship | Primary Care |
| Kalwani et al. 2021 [43] | Patients split by age, gender and baseline clinical conditions | Health Inequalities Patient experience | Preventive cardiology |
| Vongsachang et al. 2021 [44] | Children | Clinician-Patient relationship  Health Inequalities | Pediatric ophthalmology |
| Gray et al. 2021 [45] | Not specified - assume general population | Patient experience | Emergency Department |
| Tai-Seale et al. 2021 [46] | Not specified - assume general population | Clinician-Patient relationship | Ambulatory care |
| Maher et al. 2021 [47] | Not specified - assume general population | Patient Experience | Outpatient care |
| Kamimura et al. 2021 [48] | Insurance status and spoken language | Health Inequalities Patient experience | Primary care |
| Altulaihi et al. 2021 [49] | Patients split by gender | Clinician-Patient relationship | Primary Care |
| Utley et al. 2021 [50] | Not specified - assume general population | Health inequalities | Family medicine |
| Alhajri et al. 2021 [51] | Not specified - assume general population | Clinician-Patient relationship | Outpatient care |
| LeBrun et al. 2021 [52] | Not specified - assume general population | Patient experience | Outpatient Arthroplasty |
| Florea et al. 2021 [53] | Not specified - assume general population | Clinician-Patient relationship  Patient Experience | Primary Care |
| Frank et al. 2021 [54] | Low-incom adults and children with a particular condition | Health Inequalities | Mental Health |
| Orrange et al. 2021 [55] | Urban adults, split by income and age | Clinician-Patient relationship  Patient Experience | Internal Medicine |
| Kletecka-Pulker et al. 2021 [56] | Not specified - assume general population | Patient experience | Outpatient care |
| Alharbi et al. 2021 [57] | Patients split by gender and level of education | Health Inequalities Patient experience | Family medicine, obstetrics and gynecology |
| Jhaveri et al. 2022 [58] | Urban/Suburban/Rural | Clinician-Patient relationship | Rheumatology |
| Rosler 2020 [59] | Not specified - assume general population | Clinician-Patient relationship  Health Inequalities  Patient Experience | Pediatrics |
| Dhahri et al. 2020 [60] | Patients split by age | Clinician-Patient relationship  Patient Experience | Outpatient care |
| Kumar et al. 2020 [61] | Not specified - assume general population | Clinician-Patient relationship  Patient Experience | Orthopedics |
| Troncone et al. 2022 [62] | Children | Clinician-Patient relationship  Health Inequalities  Patient Experience | Primary Care |
| Assenza et al. 2020 [63] | Patients split by gender | Patient experience | Outpatient care |
| White et al. 2022 [64] | Not specified - assume general population | Clinician-Patient relationship | Primary Care |
| Villalobos and Calvanese 2021 [65] | Not specified - assume general population | Clinician-Patient relationship | Outpatient care |
| Massaroni et al. 2021 [66] | Patients with a particular condition living in a particular region split by age | Clinician-Patient relationship  Patient experience  Health inequalities | Outpatient care |
| Curtis et al. 2021 [67] | Patients split by age, gender, ethnicity and socioeconomic group | Patient experience | Primary Care |
| Mathew et al. 2021 [68] | Not specified - assume general population | Patient experience | Primary Care |
| Skelly et al. 2021 [69] | Patients distant from hospital, with mobility or chronic illness, with limited access to transport or those who require chaperoning | Clinician-Patient relationship  Patient experience  Health inequalities | Primary care and outpatient |
| Riedel et al. 2021 [70] | Insured/uninsured patients | Clinician-Patient relationship  Health Inequalities | Substance misuse |
| Moore et al. 2022 [71] | Patients split by age, gender, care giving status, education, socioeconomic status, ethnicity | Health Inequalities Patient experience | Internal Medicine |
| Ennis et al. 2021 [72] | Women and maternity status | Clinician-patient relationship | Sexual Medicine |
| Conway et al. 2021 [73] | Not specified - assume general population | Clinician-Patient relationship  Patient Experience | Neuro-ophthalmology |
| Verma and Kerrison 2022 [74] | Not specified - assume general population | Clinician-Patient relationship  Patient Experience | Primary Care |
| Kludacz-Alessandri et al. 2021 [75] | Not specified - assume general population | Clinician-Patient relationship  Patient Experience | Primary Care |
| Adams et al. 2021 [76] | Patients split by gender, age, educational qualification, health literacy and internet access | Health Inequalities Patient experience | Rheumatology |
| Franzosa et al. 2021 [77] | Older adults (with/without sensory and cognitive difficulties), access to technology, reliance on caregivers/aides | Clinician-Patient relationship | Primary Care |
| Teng et al. 2021 [78] | Not specified - assume general population | Clinician-Patient relationship  Patient Experience | Outpatient care |
| Reicher et al. 2021 [79] | Adults, using social media networks split by gender and with/without chronic conditions | Health Inequalities Patient experience | Primary care |
| Chiang et al. 2021 [80] | Not specified - assume general population | Patient experience | Primary care and outpatient |
| Javanparast et al. 2021 [81] | Population groups with low digital literacy | Clinician-Patient relationship  Patient experience  Health inequalities | Primary care |
| Gupta et al. 2021 [82] | Not specified - assume general population | Patient experience | ENT |
| Day et al. 2021 [83] | Not specified - assume general population | Patient experience | Primary Care |
| Staffieri et al. 2021 [84] | Children | Health Inequalities Patient experience | Pediatric Ophthalmology |
| Chesnel et al. 2021 [85] | Not specified - assume general population | Clinician-Patient relationship  Patient Experience | Neurology |
| Kurotschka et al. 2021 [86] | Not specified - assume general population | Clinician-Patient relationship | Primary Care |
| Malliaras et al. 2021 [87] | Not specified - assume general population | Clinician-Patient relationship | Physiotherapy |
| Krasovsky et al. 2021 [88] | Children | Clinician-Patient relationship  Health Inequalities | Pediatric Telerehabilitation |
| Mustafa et al. 2021 [89] | Not specified - assume general population | Clinician-Patient relationship  Patient Experience | Immunology |
| Franzosa et al. 2021 [90] | Elderly patients | Clinician-Patient relationship | Geriatric Home-based primary care practices |
| Saiyed et al. 2021 [91] | Not specified - assume general population | Clinician-Patient relationship | Ambulatory care |
| Kirby et al. 2021 [92] | Patients split by their ability to adopt new technology and communication effectiveness | Clinician-Patient relationship  Patient Experience | Sports Medicine |
| Khan et al. 2021 [93] | Patients split by gender, education and age. | Patient experience | Orthopedics, ophthalmology, general medicine |
| Reynolds-Wright et al. 2021 [94] | Not specified - assume general population | Clinician-Patient relationship | Sexual Medicine |
| Murren-Boezem et al. 2021 [95] | Children | Clinician-Patient relationship  Health Inequalities  Patient Experience | Pediatrics |
| Barkai et al. 2021 [96] | Not specified - assume general population | Clinician-Patient relationship  Patient experience | Outpatient care |
| Miller et al. 2021 [97] | Not specified - assume general population | Clinician-Patient relationship  Patient Experience | Physical therapy |
| Mills et al. 2021 [98] | Patients split by ethnicity, gender, income level. | Health inequalities | Primary care |
| Byrne and Watkinson 2021 [99] | Not specified - assume general population | Clinician-Patient relationship  Patient Experience | Orthodontics |
| Itamura et al. 2021 [100] | Not specified - assume general population | Patient experience | Otolaryngology |
| Kato-Lin et al. 2021 [101] | Adults, split by gender, level of internet use and education level | Health Inequalities Patient experience | Acute care |
| Holtz 2021 [102] | Patients registered with a primary care physician | Patient experience | Primary Care |
| Imlach et al. 2020 [103] | Patients with social media access | Patient experience | Primary Care |
| Schweiberger et al. 2020 [104] | Children | Health Inequalities Patient experience | Pediatric primary care |
| Berg et al. 2020 [105] | Adults | Clinician-Patient relationship  Patient experience  Health inequalities | Sexual Medicine |
| Dietzen et al. 2020 [106] | Not specified - assume general population | Clinician-Patient relationship  Health Inequalities | Pediatrics |
| Isautier et al. 2020 [107] | Children | Health Inequalities Patient experience | Pediatric Physiatry |
| Esper et al. 2020 [108] | Not specified - assume general population | Clinician-Patient relationship  Patient Experience | Outpatient care |
| Shachak et al. 2020 [109] | Not specified - assume general population | Clinician-Patient relationship  Patient Experience | Outpatient care |
| Dooley et al. 2020 [110] | Not specified - assume general population | Clinician-Patient relationship | Sexual Medicine |
| Olayiwola et al. 2020 [111] | Not specified - assume general population | Clinician-Patient relationship | Family medicine |
| Patel et al. 2020 [112] | Not specified - assume general population | Clinician-Patient relationship  Patient Experience | Outpatient care |
| Tenforde et al. 2020 [113] | Not specified - assume general population | Clinician-Patient relationship  Patient Experience | Outpatient Sports and Musculoskeletal Medicine Physicians |
| Verduzco-Gutierrez et al. 2020 [114] | Not specified - assume general population | Clinician-Patient relationship | Outpatient telerehabilitation or prerehabilitation |
| Webber et al. 2021 [115] | Urban population split by socioeconomic status | Health inequalities | Primary Care |
| Scherer et al. 2021 [116] | Patients split by age and gender | Clinician-Patient relationship  Patient experience  Health inequalities | Orthopedic and trauma care |
| Parker and Chia 2021 [117] | Patients split by age and gender | Clinician-Patient relationship  Patient experience  Health inequalities | Dentistry |
| Fluhr et al. 2021 [118] | Patients split by age and gender, as well as distance between clinic and home | Clinician-Patient relationship  Patient Experience | Dermatology |
| Lewis et al. 2021 [119] | Patients with mental health conditions | Patient experience | Outpatient care |
| Tozour et al. 2021 [120] | Women and maternity status | Patient experience | Obstetrics |
| Menhadji et al. 2021 [121] | Not specified - assume general population | Clinician-Patient relationship  Patient Experience | Dentistry |
| Kumar et al. 2021 [122] | Patients split by age, gender and level of education. | Health Inequalities Patient experience | Haematology |
| Grosman-Dziewiszek et al. 2021 [123] | Not specified - assume general population | Patient experience | Community Pharmacy |
| Dopelt et al. 2021 [124] | Patients split by level of internet use, ehealth literacy, age and education level | Health Inequalities Patient experience | Primary care |
| Wilson et al. 2021 [125] | Not specified - assume general population | Clinician-Patient relationship | Primary Care |
| Gold et al. 2021 [126] | Not specified - assume general population | Clinician-Patient relationship | Primary Care |
| Johnsen et al. 2021 [127] | Not specified - assume general population | Clinician-Patient relationship | Primary Care |
| Esade Creapolis 2020 [128] | Not specified - assume general population | Clinician-Patient relationship Patient Experience | Primary and community Care |
| Lopez et al. 2021 [129] | Legal/Illegal immigrants, socioeconomic deprivation, migrant population groups | Health Inequalities | Primary Care |
| Murillo Lesmes 2021 [130] | Not specified - assume general population | Clinician-Patient relationship  Patient Experience | Endocrinology and nutrition |
| Sutton Cherem 2021 [131] | Urban/Rural | Clinician-Patient relationship  Health Inequalities | Primary Care |
| Comité de ética asistencial de Atención Primaria de Bizkaia [132] | Not specified - assume general population | Clinician-Patient relationship  Patient Experience | Primary Care, Dermatology, pediatrics, digestive, gynecology, psychological assistance (specialties given as example in the commentary) |
| Organizacion Medica Colegial de España 2021 [133] | Urban/Rural | Clinician-Patient relationship | Primary Care |
| Kludacz-Alessandri et al. 2021 [134] | Not specified - assume general population | Patient experience | Primary Care |

**Table S2. Summary document characteristics**

|  | **Document characteristics** | **Number of documents** |
| --- | --- | --- |
|  |  |  |
| **Academic/other documents** |  |  |
|  | General Google Search | 14 |
|  | Academic study | 2 |
|  | Other document | 12 |
|  | Academic Database | 120 |
|  | Academic study | 95 |
|  | Other document | 25 |
|  |  |  |
| **Researcher location** |  |  |
|  | North America | 58 |
|  | Europe | 39 |
|  | Australia and New Zealand | 14 |
|  | Asia | 13 |
|  | South America | 3 |
|  | Africa | 2 |
|  | Not reported | 5 |
| **Telehealth Medium** |  |  |
|  | Not reported | 77 |
|  | Video | 30 |
|  | Telephone and video | 14 |
|  | Telephone | 13 |
| **Survey/Questionnaire Sample size** |  |  |
|  | 9-229 | 30 |
|  | 230-249 | 14 |
|  | 450-669 | 7 |
|  | 670-889 | 3 |
|  | 890-999 | 2 |
|  | 1,000-2,225 | 8 |
|  | 2,226+ | 3 |
|  | Not specified | 14 |
| **Methodology** |  |  |
|  | Survey | 52 |
|  | Questionnaire | 29 |
|  | Commentary | 19 |
|  | Interview | 7 |
|  | Rapid review | 5 |
|  | Questionnaire+interview | 4 |
|  | Survey+interview | 4 |
|  | Operational data analysis | 3 |
|  | Guidance | 2 |
|  | Systematic Review | 2 |
|  | Board Report | 1 |
|  | Case study | 2 |
|  | Conversation Analysis and Sociolinguistic Discourse Analysis | 1 |
|  | Local government report/plan | 1 |
|  | Randomised Controlled Trial | 1 |
|  | Survey and clinical data analysis | 1 |
| **Design** |  |  |
|  | Cross-sectional | 97 |
|  | Longitudinal | 12 |
|  | Randomised trial | 1 |
|  | Not reported | 2 |
|  | Not applicable | 22 |

Legend: The table outlines the breakdown of documents covering the various areas. For instance, 14 documents were found via Google Search, or 97 documents had a cross-sectional design.

Table S3. Summary results against protocol’s Population Context Concept

|  |  | **Protocol PCC** | **Number of texts** |
| --- | --- | --- | --- |
|  |  |  |  |
| **Population** |  |  |  |
|  | **UK protected characteristics** |  |  |
|  |  | Age^a^ | 39 |
|  |  | Sex | 20 |
|  |  | Race (or ethnicity) | 9 |
|  |  | Pregnancy and maternity | 2 |
|  |  | Disability | 2 |
|  | **Other UK protected characteristics ^b^** |  | 0 |
|  | **Other characteristics** |  |  |
|  |  | Location ^c^ | 6 |
|  |  | Socioeconomic status ^d^ | 28 |
|  |  | Digital ^e^ | 10 |
|  |  | Health status ^f^ | 12 |
|  |  | No split | 66 |
| **Context** |  |  |  |
|  |  | Outpatient specialty | 56 |
|  |  | Primary care only | 46 |
|  |  | Outpatient care | 19 |
|  |  | Primary care and outpatient | 6 |
|  |  | Emergency Department | 3 |
|  |  | Primary care and other specialties | 2 |
|  |  | Community Pharmacy | 1 |
|  |  | Various | 1 |
| **Concept** |  |  |  |
|  | **One Concept** |  |  |
|  |  | Clinician-Patient relationship | 28 |
|  |  | Health inequalities | 11 |
|  |  | Patient experience | 26 |
|  | **Two concepts** |  |  |
|  |  | Clinician-Patient relationship  Health Inequalities | 11 |
|  |  | Clinician-Patient relationship  Patient Experience | 29 |
|  |  | Health Inequalities  Patient experience | 16 |
|  | **All three concepts** |  |  |
|  |  | Clinician-Patient relationship  Patient experience  Health inequalities | 13 |

Note: The total for population adds to more than 134 as a particular document can cover more than one category. ^a^ Children, adults, elderly population. ^b^ Gender reassignment, marriage/civil partnership, religion or belief and sexual orientation. ^c^ Urban/rural or care home residence or closeness to health centre. ^d^ Education, income, insured/uninsured status, migrant, ability to speak local language, access to transport, care giving status. ^e^ Ownership, use, literacy, ability to adopt new technology. ^f^ Particular condition, chronic condition, health literacy.

Legend: The table outlines the breakdown of documents covering the elements related to the population, concept and context. For example of the 134 documents, 39 had specific references to the age of the participants in the study.

**References**

1. East Kent Hospitals NHS Foundation Trust. 2021. Board of Directors Meeting - Open (Thursday 27 May 2021) [accessed 2024-12-5] <https://www.ekhuft.nhs.uk/about-us/board-of-directors/>
2. European Commission. 2020 Nov 25. The organisation of resilient health and social care following the COVID-19 pandemic [accessed 2022-10-26] <https://health.ec.europa.eu/system/files/2020-12/026_health_socialcare_covid19_en_0.pdf>
3. Karacabeyli D, Shea S, Keidar S, Pinkney S, Bepple K, Edwards D, et al. The Live 5-2-1-0 Toolkit for family physicians: Mixed methods evaluation of a resource to facilitate health promotion in a primary care setting. BC Medical Journal. 2020;62(6):1.
4. General practice in the post Covid world: Challenges and opportunities for general practice. Royal College of General Practitioners. URL: https://www.rcgp.org.uk/getmedia/4a241eec-500b-44f7-96fe-0e63208f619b/ general-practice-post-covid-rcgp.pdf [accessed 2024-11-06]
5. Taskforce on Telehealth Policy (TTP): Findings and Recommendations. National Committee for Quality Assurance (NCQA). URL: https://www.ncqa.org/programs/data-and-information-technology/telehealth/taskforce-on-telehealth-policy/ taskforce-on-telehealth-policy-ttp-findings-and-recommendations/ [accessed 2022-10-26]
6. Focus: Artificial Intelligence and Technology in Family Medicine. Family Doctor. 2021. URL: https://www.nysafp.org/ wp-content/uploads/2024/05/Winter-2021.pdf [accessed 2024-11-06]
7. Mbunge E, Batani J, Gaobotse G, Muchemwa B. Virtual healthcare services and digital health technologies deployed during coronavirus disease 2019 (COVID-19) pandemic in South Africa: a systematic review. Glob Health J. Jun 2022;6(2):102-113.
8. Jonnagaddala J, Godinho MA, Liaw S. From telehealth to virtual primary care in Australia? A Rapid scoping review. Int J Med Inform. Jul 2021;151:104470.
9. Written evidence submitted by Philips UKI (HSC0978). UK Parliament. Mar 2021. URL: https://committees.parliament.uk/ writtenevidence/25169/pdf/ [accessed 2022-10-18]
10. Li E, Tsopra R, Jimenez G, Serafini A, Gusso G, Lingner H, et al. General practitioners' perceptions of using virtual primary care during the COVID-19 pandemic: An international cross-sectional survey study. PLOS Digit Health. May 2022;1(5):e0000029.
11. Tulupova E, Dobiášová K, Kopsa Těšinová J. Patient experience with telemedicine in primary care during the COVID pandemic: a qualitative study. European Journal of Public Health. 2021;31(Supplement_3):ckab165.162.
12. Dimer NA, do Canto-Soares N, Dos Santos-Teixeira L, de Goulart BNG. The COVID-19 pandemic and the implementation of telehealth in speech-language and hearing therapy for patients at home: an experience report. Codas. 2020;32(3):e20200144.
13. Hardcastle L, Ogbogu U. Virtual care: Enhancing access or harming care? Healthc Manage Forum. Nov 20, 2020;33(6):288-292.
14. Bergman D, Bethell C, Gombojav N, Hassink S, Stange KC. Physical Distancing With Social Connectedness. Ann Fam Med. May 01, 2020;18(3):272-277.
15. Triantafillou V, Layfield E, Prasad A, Deng J, Shanti RM, Newman JG, et al. Patient Perceptions of Head and Neck Ambulatory Telemedicine Visits: A Qualitative Study. Otolaryngol Head Neck Surg. May 14, 2021;164(5):923-931.
16. Srinivasan M, Asch S, Vilendrer S, Thomas SC, Bajra R, Barman L, et al. Qualitative Assessment of Rapid System Transformation to Primary Care Video Visits at an Academic Medical Center. Annals of Internal Medicine. Oct 06, 2020;173(7):527-535.
17. Belenkiy IG. COVID-19 Challenge: What Has Been Done and What Must Be Done? Traumatology and Orthopedics of Russia. Jul 06, 2020;26(2):15-19.
18. O'Neill C, Matias MV, Peixoto VR, O'Neill H, Aguiar P, Castela E. [Teleconsultation in the Portuguese National Health Service During the COVID-19 Pandemic: A Survey of Physicians' Views and Future Implications]. Acta Med Port. Jun 01, 2022;35(6):455-467.
19. Hofmann G, Hampanda K, Harrison MS, Fasano M, Nacht A, Yeoman M. Virtual Prenatal and Postpartum Care Acceptability Among Maternity Care Providers. Matern Child Health J. Jul 15, 2022;26(7):1401-1408.
20. Manski-Nankervis J, Davidson S, Hiscock H, Hallinan C, Ride J, Lingam V, et al. Primary care consumers’ experiences and opinions of a telehealth consultation delivered via video during the COVID-19 pandemic. Aust J Prim Health. Jun 2022;28(3):224-231.
21. Mostafa PIN, Hegazy AA. Dermatological consultations in the COVID-19 era: is teledermatology the key to social distancing? An Egyptian experience. J Dermatolog Treat. Mar 2022;33(2):910-915.
22. Ball E, Rivas C, Khan R. If virtual gynecology clinics are here to stay, we need to include everyone. AJOG Glob Rep. Feb 2022;2(1):100043.
23. Sharma AE, Khoong EC, Sierra M, Rivadeneira NA, Nijagal MA, Su G, et al. System-Level Factors Associated With Telephone and Video Visit Use: Survey of Safety-Net Clinicians During the Early Phase of the COVID-19 Pandemic. JMIR Form Res. Mar 10, 2022;6(3):e34088.
24. Grens H, de Bruin JP, Huppelschoten A, Kremer JAM. Fertility Workup With Video Consultation During the COVID-19 Pandemic: Pilot Quantitative and Qualitative Study. JMIR Form Res. Feb 07, 2022;6(2):e32000.
25. Aliberti GM, Bhatia R, Desrochers LB, Gilliam EA, Schonberg MA. Perspectives of primary care clinicians in Massachusetts on use of telemedicine with adults aged 65 and older during the COVID-19 pandemic. Prev Med Rep. Apr 2022;26:101729.
26. Rego N, Pereira HS, Crispim J. Perceptions of Patients and Physicians on Teleconsultation at Home for Diabetes Mellitus: Survey Study. JMIR Hum Factors. Nov 23, 2021;8(4):e27873.
27. Swaminathan R, Mughal Z, Phillips D. Telephone Consultation in Otorhinolaryngology During the Coronavirus Disease 2019 Pandemic: a Cross-sectional Analysis of Effectiveness and Satisfaction for Patients and Clinicians. SN Compr Clin Med. 2022;4(1):36.
28. Heyck Lee S, Ramondino S, Gallo K, Moist LM. A Quantitative and Qualitative Study on Patient and Physician Perceptions of Nephrology Telephone Consultation During COVID-19. Can J Kidney Health Dis. 2022;9:20543581211066720.
29. Olszewski C, Thomson S, Strauss L, Graham R, Ezzeddine M, Dodenhoff K, et al. Patient Experiences With Ambulatory Telehealth in Neurology: Results of a Mixed-Methods Study. Neurol Clin Pract. Dec 2021;11(6):484-496.
30. Kling SMR, Falco-Walter JJ, Saliba-Gustafsson EA, Garvert DW, Brown-Johnson CG, Miller-Kuhlmann R, et al. Patient and Clinician Perspectives of New and Return Ambulatory Teleneurology Visits. Neurol Clin Pract. Dec 2021;11(6):472-483.
31. Taylor P, Berg C, Thompson J, Dean K, Yuan T, Nallamshetty S, et al. Effective Access to Care in a Crisis Period: Hypertension Control During the COVID-19 Pandemic by Telemedicine. Mayo Clin Proc Innov Qual Outcomes. Feb 2022;6(1):19-26.
32. Tornero-Molina J, Sánchez-Alonso F, Fernández-Prada M, Bris-Ochaita M, Sifuentes-Giraldo A, Vidal-Fuentes J. Tele-Rheumatology During the COVID-19 Pandemic. Reumatol Clin (Engl Ed). Oct 29, 2020;18(3):157-163.
33. Hays RD, Skootsky SA. Patient Experience with In-Person and Telehealth Visits Before and During the COVID-19 Pandemic at a Large Integrated Health System in the United States. J Gen Intern Med. Mar 2022;37(4):847-852.
34. Thiele T, Beider S, Kühl H, Mielke G, Holz A, Hirsch S, et al. [Care of rheumatology patients during the lockdown in early 2020 : Telemedicine, delegation, patient satisfaction and vaccination behavior]. Z Rheumatol. Mar 2022;81(2):157-163.
35. Volandes AE, Zupanc SN, Paasche-Orlow MK, Lakin JR, Chang Y, Burns EA, et al. Association of an Advance Care Planning Video and Communication Intervention With Documentation of Advance Care Planning Among Older Adults: A Nonrandomized Controlled Trial. JAMA Netw Open. Feb 01, 2022;5(2):e220354.
36. Nguyen OT, Watson AK, Motwani K, Warpinski C, McDilda K, Leon C, et al. Patient-Level Factors Associated with Utilization of Telemedicine Services from a Free Clinic During COVID-19. Telemed J E Health. Apr 2022;28(4):526-534.
37. Al-Iede M, Waters K, Aleidi S, Alqutawneh B, Alnawaiseh H, Alshraideh A, et al. Impact of COVID-19 lockdown on children with asthma in Jordan: a parental questionnaire. BMJ Paediatr Open. 2021;5(1):e001136.
38. Dempsey CM, Serino-Cipoletta JM, Marinaccio BD, O'Malley KA, Goldberg NE, Dolan CM, et al. Determining factors that influence parents' perceptions of telehealth provided in a pediatric gastroenterological practice: A quality improvement project. J Pediatr Nurs. 2022;62:36-42.
39. Parsons MW, Gardner MM, Sherman JC, Pasquariello K, Grieco JA, Kay CD, et al. Feasibility and Acceptance of Direct-to-Home Tele-neuropsychology Services during the COVID-19 Pandemic. J Int Neuropsychol Soc. Feb 2022;28(2):210-215.
40. Fu L, Lindenmeyer A, Phillimore J, Lessard-Phillips L. Vulnerable migrants' access to healthcare in the early stages of the COVID-19 pandemic in the UK. Public Health. Feb 2022;203:36-42.
41. Sugarman DE, Horvitz LE, Greenfield SF, Busch AB. Clinicians' Perceptions of Rapid Scale-up of Telehealth Services in Outpatient Mental Health Treatment. Telemed J E Health. Dec 2021;27(12):1399-1408.
42. Johnson C, Dupuis JB, Goguen P, Grenier G. Changes to telehealth practices in primary care in New Brunswick (Canada): A comparative study pre and during the COVID-19 pandemic. PLoS One. 2021;16(11):e0258839.
43. Kalwani NM, Wang KM, Johnson AN, Deb JD, Gold T, Maddukuri AK, et al. Application of the Quadruple Aim to evaluate the operational impact of a telemedicine program. Healthc (Amst). Dec 2021;9(4):100593.
44. Vongsachang H, Lagstein O, Boland MV, Repka MX, Kraus CL, Collins ME. Telemedicine utilization by pediatric ophthalmologists during the COVID-19 pandemic. J AAPOS. Oct 2021;25(5):293-295.e1.
45. Gray DP, Sidaway-Lee K, Harding A, Evans P. Reduction in face-to-face GP consultations. Br J Gen Pract. Jul 2020;70(696):328.
46. Tai-Seale M, Rosen R, Ruo B, Hogarth M, Longhurst CA, Lander L, et al. Implementation of Patient Engagement Tools in Electronic Health Records to Enhance Patient-Centered Communication: Protocol for Feasibility Evaluation and Preliminary Results. JMIR Res Protoc. Aug 26, 2021;10(8):e30431.
47. Maher DP, Hess D, Edwards C, Allen L. Changes in Patient Satisfaction Scores During the Early COVID-19 Pandemic. J Patient Exp. 2021;8:23743735211034610.
48. Kamimura A, Panahi S, Meng H, Sundrud J, Lucero M. Patient Satisfaction With Telehealth and Experiences During the COVID-19 Pandemic Among Uninsured Free Clinic Patients. J Patient Exp. Jul 26, 2021;8:23743735211033107.
49. Altulaihi BA, Alharbi KG, Alhassan AM, Altamimi AM, Al Akeel MA. Physician's Perception Toward Using Telemedicine During COVID-19 Pandemic in King Abdulaziz Medical City, Riyadh, Saudi Arabia. Cureus. Jul 2021;13(7):e16107
50. Utley LM, Manchala GS, Phillips MJ, Doshi CP, Szatalowicz VL, Boozer JR. Bridging the Telemedicine Gap Among Seniors During the COVID-19 Pandemic. J Patient Exp. 2021;8:23743735211014036.
51. Alhajri N, Simsekler MCE, Alfalasi B, Alhashmi M, AlGhatrif M, Balalaa N, et al. Physicians' Attitudes Toward Telemedicine Consultations During the COVID-19 Pandemic: Cross-sectional Study. JMIR Med Inform. Jun 01, 2021;9(6):e29251.
52. LeBrun DG, Malfer C, Wilson M, Carroll KM, Wang Ms V, Mayman DJ, et al. Telemedicine in an Outpatient Arthroplasty Setting During the COVID-19 Pandemic: Early Lessons from New York City. HSS J. Feb 2021;17(1):25-30.
53. Florea M, Lazea C, Gaga R, Sur G, Lotrean L, Puia A, et al. Lights and Shadows of the Perception of the Use of Telemedicine by Romanian Family Doctors During the COVID-19 Pandemic. Int J Gen Med. 2021;14:1575-1587.
54. Frank HE, Grumbach NM, Conrad SM, Wheeler J, Wolff J. Mental health services in primary care: Evidence for the feasibility of telehealth during the COVID-19 pandemic. J Affect Disord Rep. Jul 2021;5:100146.
55. Orrange S, Patel A, Mack WJ, Cassetta J. Patient Satisfaction and Trust in Telemedicine During the COVID-19 Pandemic: Retrospective Observational Study. JMIR Hum Factors. Apr 22, 2021;8(2):e28589.
56. Kletečka-Pulker M, Völkl-Kernstock S, Fassl A, Klager E, Willschke H, Klomfar S, et al. Telehealth in Times of COVID-19: Spotlight on Austria. Healthcare (Basel). Mar 04, 2021;9(3):1.
57. Alharbi KG, Aldosari MN, Alhassan AM, Alshallal KA, Altamimi AM, Altulaihi BA. Patient satisfaction with virtual clinic during Coronavirus disease (COVID-19) pandemic in primary healthcare, Riyadh, Saudi Arabia. J Family Community Med. 2021;28(1):48-54.
58. Jhaveri D, Emeto TI, Alele FO, Strom A, Benham H. Use of telemedicine for rheumatology practice in Queensland, Australia: experiences before and during the COVID-19 pandemic. Intern Med J. Oct 2022;52(10):1685-1690.
59. Rosler G. Pediatric Telehealth Experiences: Myths and Truths About Video Visits From a Parent. J Patient Exp. Dec 2020;7(6):836-838.
60. Dhahri AA, Iqbal MR, Pardoe H. Agile Application of Video Telemedicine During the COVID-19 Pandemic. Cureus. Nov 04, 2020;12(11):e11320.
61. Kumar S, Kumar A, Kumar M, Kumar A, Arora R, Sehrawat R. Feasibility of telemedicine in maintaining follow-up of orthopaedic patients and their satisfaction: A preliminary study. J Clin Orthop Trauma. Oct 2020;11(Suppl 5):S704-S710.
62. Troncone A, Cascella C, Chianese A, Zanfardino A, Casaburo F, Piscopo A, et al. Doctor-Patient Relationship in Synchronous/Real-time Video-Consultations and In-Person Visits: An Investigation of the Perceptions of Young People with Type 1 Diabetes and Their Parents During the COVID-19 Pandemic. Int J Behav Med. Oct 2022;29(5):638-647.
63. Assenza G, Lanzone J, Brigo F, Coppola A, Di Gennaro G, Di Lazzaro V, et al. Epilepsy Care in the Time of COVID-19 Pandemic in Italy: Risk Factors for Seizure Worsening. Front Neurol. 2020;11:737.
64. White IR, Royston P, Wood AM. Multiple imputation using chained equations: Issues and guidance for practice. Stat Med. Feb 20, 2011;30(4):377-399.
65. Villalobos J, Calvanese N. [The impact of COVID-19 pandemic on doctor-patient relationship]. Rev Med Chil. Jul 2021;149(7):1070-1074.
66. Massaroni V, Delle Donne V, Ciccarelli N, Ciccullo A, Borghetti A, Faliero D, et al. Use of telehealth for HIV care in Italy: Are doctors and patients on the same page? A cross-sectional study. Int J Med Inform. Dec 2021;156:104616.
67. Curtis M, Duncan R, Jing M, Kim A, Lu VT, Redshaw J, et al. "Not a perfect situation, but..." A single-practice survey of patient experience of phone consultations during COVID-19 Alert Level 4 in New Zealand. N Z Med J. Oct 22, 2021;134(1544):35-48.
68. Mathew T, Lee PC, Ianno DJ, Benson J. Telehealth and Australian general practice in 2020: A survey exploring patients' perspectives in the Adelaide Hills. Aust J Gen Pract. Oct 2021;50(10):754-759.
69. Skelly JR, O'Connor T. Guidelines for the use of the Attend Anywhere Platform for Telecommunications within the Pain Service. Ir Med J. Aug 19, 2021;114(7):403.
70. Riedel L, Uscher-Pines L, Mehrotra A, Busch AB, Barnett ML, Raja P, et al. Use of telemedicine for opioid use disorder treatment - Perceptions and experiences of opioid use disorder clinicians. Drug Alcohol Depend. Nov 01, 2021;228:108999
71. Moore A, Chavez C, Fisher MP. Factors Enhancing Trust in Electronic Communication Among Patients from an Internal Medicine Clinic: Qualitative Results of the RECEPT Study. J Gen Intern Med. Sep 19, 2022;37(12):3121-3127.
72. Ennis M, Wahl K, Jeong D, Knight K, Renner R, Munro S, et al. The perspective of Canadian health care professionals on abortion service during the COVID-19 pandemic. Fam Pract. Aug 27, 2021;38(Suppl 1):i30-i36.
73. Conway J, Krieger P, Hasanaj L, Sun L, Scharf JM, Odel JG, et al. Telemedicine Evaluations in Neuro-Ophthalmology During the COVID-19 Pandemic: Patient and Physician Surveys. J Neuroophthalmol. Sep 01, 2021;41(3):356-361.
74. Verma P, Kerrison R. Patients' and physicians' experiences with remote consultations in primary care during the COVID-19 pandemic: a multi-method rapid review of the literature. BJGP Open. Jun 2022;6(2):1.
75. Kludacz-Alessandri M, Hawrysz L, Korneta P, Gierszewska G, Pomaranik W, Walczak R. The impact of medical teleconsultations on general practitioner-patient communication during COVID- 19: A case study from Poland. PLoS One. 2021;16(7):e0254960.
76. Adams L, Lester S, Hoon E, van der Haak H, Proudman C, Hall C, et al. Patient satisfaction and acceptability with telehealth at specialist medical outpatient clinics during the COVID-19 pandemic in Australia. Intern Med J. Jul 2021;51(7):1028-1037
77. Franzosa E, Gorbenko K, Brody AA, Leff B, Ritchie CS, Kinosian B, et al. "At Home, with Care": Lessons from New York City Home-based Primary Care Practices Managing COVID-19. J Am Geriatr Soc. Feb 2021;69(2):300-306.
78. Teng T, Sareidaki DE, Chemaly N, Bar C, Coste-Zeitoun D, Kuchenbuch M, et al. Physician and patient satisfaction with the switch to remote outpatient encounters in epilepsy clinics during the Covid-19 pandemic. Seizure. Oct 2021;91:60-65.
79. Reicher S, Sela T, Toren O. Using Telemedicine During the COVID-19 Pandemic: Attitudes of Adult Health Care Consumers in Israel. Front Public Health. 2021;9:653553.
80. Chiang C, Halker Singh R, Lalvani N, Shubin Stein K, Henscheid Lorenz D, Lay C, et al. Patient experience of telemedicine for headache care during the COVID-19 pandemic: An American Migraine Foundation survey study. Headache. May 2021;61(5):734-739.
81. Javanparast S, Roeger L, Kwok Y, Reed RL. The experience of Australian general practice patients at high risk of poor health outcomes with telehealth during the COVID-19 pandemic: a qualitative study. BMC Fam Pract. Apr 08, 2021;22(1):69.
82. Gupta T, Gkiousias V, Bhutta MF. A systematic review of outcomes of remote consultation in ENT. Clin Otolaryngol. Jul 2021;46(4):699-719.
83. Day K, Hunter I, Rao V, Russell G, Roskvist R, Moir F, et al. Survey protocol for exploring video and phone use in Aotearoa New Zealand general practice: considerations for future telehealth. BMJ Health Care Inform. Mar 2021;28(1):1.
84. Staffieri SE, Mathew AA, Sheth SJ, Ruddle JB, Elder JE. Parent satisfaction and acceptability of telehealth consultations in pediatric ophthalmology: initial experience during the COVID-19 pandemic. J AAPOS. Apr 2021;25(2):104-107.
85. Chesnel C, Hentzen C, Le Breton F, Turmel N, Tan E, Haddad R, et al. Efficiency and satisfaction with telephone consultation of follow-up patients in neuro-urology: Experience of the COVID-19 pandemic. Neurourol Urodyn. Mar 2021;40(3):929-937.
86. Kurotschka PK, Serafini A, Demontis M, Serafini A, Mereu A, Moro MF, et al. General Practitioners' Experiences During the First Phase of the COVID-19 Pandemic in Italy: A Critical Incident Technique Study. Front Public Health. 2021;9:623904.
87. Malliaras P, Merolli M, Williams CM, Caneiro JP, Haines T, Barton C. 'It's not hands-on therapy, so it's very limited': Telehealth use and views among allied health clinicians during the coronavirus pandemic. Musculoskelet Sci Pract. Apr 2021;52:102340.
88. Krasovsky T, Silberg T, Barak S, Eisenstein E, Erez N, Feldman I, et al. Transition to Multidisciplinary Pediatric Telerehabilitation during the COVID-19 Pandemic: Strategy Development and Implementation. Int J Environ Res Public Health. Feb 04, 2021;18(4):1.
89. Mustafa SS, Vadamalai K, Ramsey A. Patient Satisfaction with In-Person, Video, and Telephone Allergy/Immunology Evaluations During the COVID-19 Pandemic. J Allergy Clin Immunol Pract. May 2021;9(5):1858-1863.
90. Franzosa E, Gorbenko K, Brody AA, Leff B, Ritchie CS, Kinosian B, et al. "There Is Something Very Personal About Seeing Someone's Face": Provider Perceptions of Video Visits in Home-Based Primary Care During COVID-19. J Appl Gerontol. Nov 2021;40(11):1417-1424.
91. Saiyed S, Nguyen A, Singh R. Physician Perspective and Key Satisfaction Indicators with Rapid Telehealth Adoption During the Coronavirus Disease 2019 Pandemic. Telemed J E Health. Nov 2021;27(11):1225-1234.
92. Kirby DJ, Fried JW, Buchalter DB, Moses MJ, Hurly ET, Cardone DA, et al. Patient and Physician Satisfaction with Telehealth During the COVID-19 Pandemic: Sports Medicine Perspective. Telemed J E Health. Oct 2021;27(10):1151-1159.
93. Khan ZA, Zahoor A, Afzal I, Butt U, Siddiqui AM, Khan Z, et al. Evaluation of Patient Perception and Satisfaction Toward the Use of Telemedicine During Pandemic of Novel Coronavirus in Pakistan. Telemed J E Health. Oct 2021;27(10):1174-1179.
94. Reynolds-Wright JJ, Boydell N, Cameron S, Harden J. A qualitative study of abortion care providers' perspectives on telemedicine medical abortion provision in the context of COVID-19. BMJ Sex Reprod Health. Jul 2022;48(3):199-204.
95. Murren-Boezem J, Solo-Josephson P, Zettler-Greeley CM. On-Demand, Virtual Health Care During COVID-19: Clinician Redeployment and Telemedicine Utilization in a Children's Health System. Telemed J E Health. Oct 2021;27(10):1111-1116
96. Barkai G, Gadot M, Amir H, Menashe M, Shvimer-Rothschild L, Zimlichman E. Patient and clinician experience with a rapidly implemented large-scale video consultation program during COVID-19. Int J Qual Health Care. Feb 20, 2021;33(1):1.
97. Miller MJ, Pak SS, Keller DR, Barnes DE. Evaluation of Pragmatic Telehealth Physical Therapy Implementation During the COVID-19 Pandemic. Phys Ther. Jan 04, 2021;101(1):1.
98. Mills KT, Peacock E, Chen J, Zimmerman A, He H, Cyprian A, et al. Experiences and Beliefs of Low-Income Patients With Hypertension in Louisiana and Mississippi During the COVID-19 Pandemic. J Am Heart Assoc. Feb 02, 2021;10(3):e018510.
99. Byrne E, Watkinson S. Patient and clinician satisfaction with video consultations during the COVID-19 pandemic: an opportunity for a new way of working. J Orthod. Mar 2021;48(1):64-73.
100. Itamura K, Tang DM, Higgins TS, Rimell FL, Illing EA, Ting JY, et al. Comparison of Patient Satisfaction Between Virtual Visits During the COVID-19 Pandemic and In-person Visits Pre-pandemic. Ann Otol Rhinol Laryngol. Jul 2021;130(7):810-817.
101. Kato-Lin Y, Thelen ST. Telemedicine for Acute Conditions During COVID-19: A Nationwide Survey Using Crowdsourcing. Telemed J E Health. Jul 2021;27(7):714-723.
102. Holtz BE. Patients Perceptions of Telemedicine Visits Before and After the Coronavirus Disease 2019 Pandemic. Telemed J E Health. Jan 01, 2021;27(1):107-112.
103. Imlach F, McKinlay E, Middleton L, Kennedy J, Pledger M, Russell L, et al. Telehealth consultations in general practice during a pandemic lockdown: survey and interviews on patient experiences and preferences. BMC Fam Pract. Dec 13, 2020;21(1):269.
104. Schweiberger K, Hoberman A, Iagnemma J, Schoemer P, Squire J, Taormina J, et al. Practice-Level Variation in Telemedicine Use in a Pediatric Primary Care Network During the COVID-19 Pandemic: Retrospective Analysis and Survey Study. J Med Internet Res. Dec 18, 2020;22(12):e24345.
105. Berg WT, Goldstein M, Melnick AP, Rosenwaks Z. Clinical implications of telemedicine for providers and patients. Fertil Steril. Dec 2020;114(6):1129-1134.
106. Dietzen A, Ide W, Pavone L. Telehealth in pediatric rehabilitation medicine: A survey of clinician reported utilization and experience. J Pediatr Rehabil Med. 2020;13(3):281-288.
107. Isautier JM, Copp T, Ayre J, Cvejic E, Meyerowitz-Katz G, Batcup C, et al. People's Experiences and Satisfaction With Telehealth During the COVID-19 Pandemic in Australia: Cross-Sectional Survey Study. J Med Internet Res. Dec 10, 2020;22(12):e24531.
108. Esper GJ, Sweeney RL, Winchell E, Duffell JM, Kier SC, Lukens HW, et al. Rapid Systemwide Implementation of Outpatient Telehealth in Response to the COVID-19 Pandemic. J Healthc Manag. 2020;65(6):443-452.
109. Shachak A, Alkureishi MA. Virtual care: a 'Zoombie' apocalypse? J Am Med Inform Assoc. Nov 01, 2020;27(11):1813-1815.
110. Dooley AB, Houssaye NDL, Baum N. Use of Telemedicine for Sexual Medicine Patients. Sex Med Rev. Oct 2020;8(4):507-517.
111. Olayiwola JN, Magaña C, Harmon A, Nair S, Esposito E, Harsh C, et al. Telehealth as a Bright Spot of the COVID-19 Pandemic: Recommendations From the Virtual Frontlines ("Frontweb"). JMIR Public Health Surveill. Jun 25, 2020;6(2):e19045.
112. Patel S, Douglas-Moore J. A reflection on an adapted approach from face-to-face to telephone consultations in our Urology Outpatient Department during the COVID-19 pandemic - a pathway for change to future practice? BJU Int. Sep 04, 2020;126(3):339-341.
113. Tenforde AS, Iaccarino MA, Borgstrom H, Hefner JE, Silver J, Ahmed M, et al. Telemedicine During COVID-19 for Outpatient Sports and Musculoskeletal Medicine Physicians. PM R. Sep 2020;12(9):926-932.
114. Verduzco-Gutierrez M, Bean AC, Tenforde AS, Tapia RN, Silver JK. How to Conduct an Outpatient Telemedicine Rehabilitation or Prehabilitation Visit. PM R. Jul 07, 2020;12(7):714-720.
115. Webber EC, McMillen BD, Willis DR. Health Care Disparities and Access to Video Visits Before and After the COVID-19 Pandemic: Findings from a Patient Survey in Primary Care. Telemed J E Health. May 2022;28(5):712-719.
116. Scherer J, Back DA, Thienemann F, Kaufmann E, Neuhaus V, Willy C, et al. The effect of Covid-19 on the willingness to use video consultations among orthopedic and trauma outpatients: a multi-center survey in 1400 outpatients. Eur J Trauma Emerg Surg. Jun 2022;48(3):2199-2206.
117. Parker K, Chia M. Patient and clinician satisfaction with video consultations in dentistry - part one: patient satisfaction. Br Dent J. May 27, 2021:1-6.
118. Fluhr JW, Gueguen A, Legoupil D, Brenaut E, Abasq C, Araújo H, et al. Teledermatology in Times of COVID-19 Confinement: Comparing Patients' and Physicians' Satisfaction by the Standardized Brest Teledermatology Questionnaire. Dermatology. Feb 10, 2021;237(2):1-6.
119. Lewis YD, Elran-Barak R, Grundman-Shem Tov R, Zubery E. The abrupt transition from face-to-face to online treatment for eating disorders: a pilot examination of patients' perspectives during the COVID-19 lockdown. J Eat Disord. Mar 05, 2021;9(1):31.
120. Tozour JN, Bandremer S, Patberg E, Zavala J, Akerman M, Chavez M, et al. Application of telemedicine video visits in a maternal-fetal medicine practice at the epicenter of the COVID-19 pandemic. Am J Obstet Gynecol MFM. Nov 2021;3(6):100469.
121. Menhadji P, Patel R, Asimakopoulou K, Quinn B, Khoshkhounejad G, Pasha P, et al. Patients' and dentists' perceptions of tele-dentistry at the time of COVID-19. A questionnaire-based study. J Dent. Oct 2021;113:103782.
122. Kumar P, Aggarwal M, Dhawan R, Dass J, Kumar G, Sharma V, et al. Tele-Medicine Services in Hematological Practice During Covid Pandemic: Its Feasibility and Difficulties. Indian J Hematol Blood Transfus. Oct 2021;37(4):528-533.
123. Grosman-Dziewiszek P, Wiatrak B, Jęśkowiak I, Szeląg A. Patients' Habits and the Role of Pharmacists and Telemedicine as Elements of a Modern Health Care System during the COVID-19 Pandemic. J Clin Med. Sep 17, 2021;10(18):4211.
124. Dopelt K, Avni N, Haimov-Sadikov Y, Golan I, Davidovitch N. Telemedicine and eHealth Literacy in the Era of COVID-19: A Cross-Sectional Study in a Peripheral Clinic in Israel. Int J Environ Res Public Health. Sep 10, 2021;18(18):9556.
125. Wilson G, Windner Z, Bidwell S, Currie O, Dowell A, Halim AA, et al. ‘Here to stay’: changes to prescribing medication in general practice during the COVID-19 pandemic in New Zealand. J. Prim. Health Care. Aug 13, 2021;13(3):222-230.
126. Gold KJ, Laurie AR, Kinney DR, Harmes KM, Serlin DC. Video Visits: Family Physician Experiences With Uptake During the COVID-19 Pandemic. Fam Med. Mar 4, 2021;53(3):207-210.
127. Johnsen TM, Norberg BL, Kristiansen E, Zanaboni P, Austad B, Krogh FH, et al. Suitability of Video Consultations During the COVID-19 Pandemic Lockdown: Cross-sectional Survey Among Norwegian General Practitioners. J Med Internet Res. Feb 08, 2021;23(2):e26433.
128. Estudio sobre la consulta de salud virtual y sus beneficios para el sistema sanitario. EsadeGov. Jul 16, 2020. URL: https:/ /www.esade.edu/faculty-research/es/esadegov/media/
129. Lopez Guzman Y, Barros Castillo SM, Leal Betancourt MI. Condiciones de vulnerabilidad, sociodemográficas y clínicas de la población con COVID-19 incluida en la estrategia de atención primaria en salud. Mosquera, 2020. Universidad del Rosario. Oct 2021. URL: https://repository.urosario.edu.co/bitstream/handle/10336/33058/LopezGuzman-YudiCarolina-2021. pdf?sequence=6&isAllowed=y [accessed 2022-10-26]
130. Gorgojo Martínez JJ, Murillo AZ, Rubio Herrera MÁ, Lesmes IB. TELECONSULTA en Endocrinología y Nutrición en tiempos de la pandemia COVID-19 y más allá. Sociedad Española de Endocrinología y Nutrición. URL: https://www. seen.es/ModulGEX/workspace/publico/modulos/web/docs/apartados/1433/160620_105727_7128864936.pdf [accessed 2024-11-06]
131. Hamui-Sutton L, Halabe-Cherem J. Videoconsultas: alcances y limitaciones. Medicina Interna de México. 2021;37(2):261-266.
132. La Telemedicina Cara a Cara. Comité de ética asistencial de Atención Primaria de Bizkaia. Dec 2020. URL: https://osalde. org/wp-content/uploads/2021/01/Telemedicina-aspectos-%C3%A9ticos-CEAAPB.pdf [accessed 2022-10-26]
133. La Atención Primaria Rural en España lanza un grito de socorro para su supervivencia. Médicos y Pacientes. URL: http:/ /www.medicosypacientes.com/articulo/la-atencion-primaria-rural-en-espana-lanza-un-grito-de-socorro-para-su-supervivencia [accessed 2022-11-05]
134. Kludacz-Alessandri M, Walczak R, Hawrysz L, Korneta P. The Quality of Medical Care in the Conditions of the COVID-19 Pandemic, with Particular Emphasis on the Access to Primary Healthcare and the Effectiveness of Treatment in Poland. J Clin Med. Aug 09, 2021;10(16):3502.
